# Comparative Evaluation of *Undaria pinnatifida* Anatomical Parts for Skin-Related Functional Properties in UVB-Irradiated L929 Fibroblasts Under Normal and High-Glucose Culture Conditions

**DOI:** 10.3390/foods15193571

**Published:** 2026-10-08

**Authors:** Rea Mae Cuario Templonuevo, Yue Zhao, Jiyeon Chun

**Affiliations:** 1 Department of Food Science and Technology, Sunchon National University, Suncheon 57922, Jeonnam, Republic of Korea; reamaetemplonuevo@clsu.edu.ph (R.M.C.T.); zy20220405@gmail.com (Y.Z.); 2 College of Fisheries-Freshwater Aquaculture Center, Central Luzon State University, Science City of Muñoz, Nueva Ecija 3120, Philippines; 3 Bio-Healthcare Research and Analysis Center, Sunchon National University, Suncheon 57922, Jeonnam, Republic of Korea

**Keywords:** *Undaria pinnatifida*, sea mustard by-products, skin-related functional properties, oxidative stress, fibroblasts, UVB irradiation

## Abstract

Sea mustard (*Undaria pinnatifida*, UP) is widely cultivated for food in many countries, including South Korea, but several anatomical parts, including the sporophyll, root, and stem, remain underutilized and represent potential resources for value-added utilization. This study comparatively evaluated the in vitro biological activities of extracts from the UP leaf and underutilized anatomical parts, with particular emphasis on skin-related functional properties in UVB-irradiated L929 fibroblasts cultured under normal- and high-glucose conditions. Inhibitory activities against skin-related enzymes were assessed using tyrosinase, hyaluronidase, elastase, and collagenase assays, together with α-glucosidase and xanthine oxidase inhibition. The root extract exhibited the strongest enzyme inhibitory activities, including tyrosinase (35.58%), hyaluronidase (64.58%), elastase (10.05%), and collagenase (98.04%), and showed the lowest IC_50_ values for α-glucosidase (0.22 mg/mL) and xanthine oxidase (0.65 μg/mL). At 50 μg/mL, all extracts maintained relative MTS metabolic activity above that of the untreated control, while live/dead staining indicated low percentages of dead cells (9.75–15.89%). UP extracts reduced intracellular ROS under the tested oxidative-stress conditions, with the root extract generally showing the greatest reduction, and promoted in vitro scratch closure relative to untreated cells. These findings demonstrate the in vitro functional potential of different *U. pinnatifida* anatomical parts, particularly the underutilized root, and support their further investigation as value-added functional ingredients.

## 1. Introduction

Skin is the largest organ in the human body, which is directly exposed to different environmental conditions. One of the causes of skin problems is the UV radiation emitted by the sun. Prolonged UV exposure, particularly UVB, can cause skin inflammation, photoaging, and even skin cancer [1]. It can potentially generate genomic lesions in the nuclear and mitochondrial DNA or produce reactive oxygen species (ROS) that elevate oxidative stress and damage cellular components [2]. According to Jesumani et al. [3], UVB exposure is known to be the primary cause of non-melanoma skin cancer.

Skin problems are also common for diabetic patients, specifically bacterial and fungal infections, affecting nearly 70% of the total diabetic population [4]. Hyperglycemia disrupts skin homeostasis by inhibiting keratinocyte and fibroblast function, impairing wound healing, and causing vascular dysfunction [5]. Common symptoms include dryness, darkening, thickening, and ulceration, often leading to severe complications like amputations.

Nowadays, the demand for several skin products has risen, especially in incorporating natural ingredients in cosmetics. This shift is driven by the environmental benefits and reduced side effects associated with natural formulations [6]. In contrast, synthetic chemicals traditionally used in cosmetic production are often costly and linked to adverse effects, including skin dermatitis and potential carcinogenicity with prolonged use [7]. The global market value of natural cosmetics derived from plants and minerals is projected to reach USD 54.5 billion by 2027 [8].

Seaweed has traditionally been used as food or a food ingredient, but its application in health and cosmetic products is expanding due to its rich functional compounds. These include carbohydrates, phenols, natural pigments, sterols, proteins, peptides, vitamins, and minerals, which exhibit anti-diabetic, anti-obesity, and anti-inflammatory properties [9]. Jia et al. [10] found that seaweed-derived polysaccharides help mitigate hyperglycemia, hyperlipidemia, and liver and kidney damage in diabetic rats. Additionally, primary and secondary metabolites from seaweed enhance skin moisture, regulate blood flow, and promote cellular renewal [11]. For instance, Jesumani et al. [3] demonstrated that polysaccharides extracted from Sargassum species exhibit anti-wrinkle and skin-whitening properties.

Sea mustard (*Undaria pinnatifida*) is a brown seaweed largely cultivated in South Korea. In 2022, about 585,955 metric tons of sea mustard were produced in the country [12], mainly for food and animal feed [13]. According to Peñalver et al. [14], *U. pinnatifida* contains several beneficial compounds and can be potentially used as a functional ingredient in a wide range of technological applications to create functional foods. The commonly utilized part of sea mustard is the leaf, whereas other anatomical parts, including the sporophyll, root, and stem, remain underutilized.

Although several biological activities of *U. pinnatifida* and its isolated bioactive constituents have been reported, comparatively limited information is available on the skin-related biological activities of its underutilized anatomical parts. In particular, comparative evaluation of the leaf, sporophyll, root, and stem under UVB-induced oxidative stress and high-glucose culture conditions remains limited. Because both UVB exposure and high-glucose environments are associated with increased oxidative stress and impaired fibroblast function, evaluation under these conditions may provide additional information on the biological responses of the different *U. pinnatifida* fractions.

Therefore, this study aimed to comparatively evaluate 80% ethanol extracts obtained from the leaf and three underutilized parts (sporophyll, root, and stem) of *U. pinnatifida*. Their enzyme inhibitory activities were assessed using cell-free assays, followed by evaluation of cellular metabolic activity, intracellular ROS levels, and scratch-wound closure in murine L929 fibroblasts under non-irradiated and UVB-irradiated conditions in normal- and high-glucose culture media. This study sought to identify potentially valuable *U. pinnatifida* fractions for further investigation as sustainable functional ingredients while providing a basis for the valorization of underutilized seaweed resources.

## 2. Materials and Methods

### 2.1. Materials and Reagents

Standard chemicals including L-tyrosine, arbutin, N-Succinyl-Ala-Ala-Ala-p-nitroanilide, Tris hydrochloride, N-[3-(2-Furyl) acryloyl]-Leu-Gly-Pro-Ala, 5 mM p-Nitrophenyl-α-D-glucopyranoside, α-glucosidase enzyme from Saccharomyces cerevisiae, acarbose, and Reactive Oxygen Species (ROS) Fluorometric Assay Kit EEA019 were all purchased from Sigma-Aldrich (St. Louis, MO, USA), along with the enzymes mushroom tyrosinase, bovine testes hyaluronidase, porcine pancreas elastase, and *Clostridium histolyticum* collagenase. Calcium carbonate and sodium borate were both obtained from Daejung Chemicals (Siheung-si, Gyeonggi-do, Republic of Korea), while L-ascorbic acid was acquired from Junsei Chemical (Tokyo, Japan). The NCTC clone 929 (L cell, L-929) from mice (Mus musculus) was purchased from ATCC (American Type Culture Collection), which was distributed by Koram Biotech Corp. (Seoul, Republic of Korea). Fetal Bovine Serum (FBS), RPMI 1640 (Roswell Park Memorial Institute) 1640 medium, and live/dead™ viability/cytotoxicity kit L3224 were obtained from Thermo Fisher Scientific (Waltham, MA, USA), while the CellTiter 96^®^ AQueous One Solution Cell Proliferation Assay kit was bought from Promega (Madison, WI, USA).

### 2.2. Preparation of U. pinnatifida Extracts

The *U. pinnatifida* (UP) samples were obtained from Bada & Haecho Fishery Corp (Goheung, Jeonnam, Republic of Korea), between March and May 2023. The samples included the leaf and waste parts, specifically the sporophyll, root, and stem. All samples were freeze-dried at −70 °C using a freeze dryer (Il-Shin Freeze Dryer Series, Il-Shinbiobase Co., Ltd., Yangju, Republic of Korea) and sieved to a particle size of 300 μm. Subsequently, 15 g of each sample was extracted with 80% ethanol at a solid-to-liquid ratio of 1:10 (*v*/*v*) at room temperature. The extraction was performed with continuous shaking for 24 h using a shaking incubator (Hanbaek HB-201SF, Bucheon-si, Gyeonggi-do, Republic of Korea). The extracts were concentrated using a vacuum rotary evaporator (Eyela N-1300E, Bunkyo-Ku, Tokyo, Japan) at 45 °C [15]. The same extraction procedure was applied to all anatomical parts to ensure consistent preparation of the extracts used for comparative biological evaluation.

### 2.3. Skin Enzyme Inhibitory Assay

#### 2.3.1. Tyrosinase Inhibitory Assay

The tyrosinase inhibitory activity of sea mustard extracts was evaluated using the method of Muddathir et al. [16], with minor modifications. Briefly, 70 μL of UP extract at a concentration of 1 mg/mL was added to a 96-well microplate. Subsequently, 30 μL of mushroom tyrosinase (333 units/mL) prepared in 50 mM phosphate buffer (pH 6.5) and 110 μL of 2 mM L-tyrosine were added to each well. The mixture was thoroughly mixed and incubated at 37 °C for 30 min. After incubation, the absorbance was measured at 510 nm using a microplate reader (Eon, BioTek Instruments, Winooski, VT, USA). Arbutin was used as a positive control. The inhibition activity was computed using the formula below.

Tyrosinase inhibition assay (%) = [1 − (A_se_ − A_s_)/(A_ce_ − A_c_)] × 100 (1)

where

A_ce_ = absorbance of control with enzyme

A_c_ = absorbance of control without enzyme

A_se_ = absorbance of test sample with enzyme

A_s_ = absorbance of test sample without enzyme

#### 2.3.2. Hyaluronidase Inhibitory Assay

The hyaluronidase inhibitory activity of sea mustard extracts was assessed using the method of Castejon [17], with minor modifications. One hundred microliters of UP extract at a concentration of 1 mg/mL were combined with 100 μL of type-1-S bovine testes hyaluronidase (2100 units/mL) in 0.1 M acetate buffer (pH 3.5) and incubated at 37 °C for 20 min. Next, 200 μL of 6 mM CaCl_2_ was added, and the mixture was incubated at 37 °C for an additional 20 min to activate the hyaluronidase with Ca^2+^. The activated hyaluronidase was then combined with 250 μL of sodium hyaluronate (1.2 mg/mL) in 0.1 M acetate buffer (pH 3.5) and incubated at 37 °C for 40 min. After incubation, 50 μL of 0.9 M NaOH and 100 μL of 0.2 M Na_2_B_4_O_7_ were added, followed by heating in a boiling water bath for 5 min. Subsequently, 250 μL of ρ-dimethylaminobenzaldehyde (DAMB) solution was added, and the mixture was incubated at room temperature for 45 min. Absorbance was measured at 585 nm using a microplate reader (Eon, BioTek Instruments, Winooski, VT, USA). Ascorbic acid was used as a positive control. The inhibition activity was computed using the formula below.

Hyaluronidase inhibition assay (%) = [1 − (EA_s_/EA_c_)] × 100 (2)

where

EA_s_ = enzyme activity of the sample

EA_c_ = enzyme activity of the control

#### 2.3.3. Elastase Inhibitory Assay

The elastase inhibitory activity of sea mustard extracts was evaluated using the method of Tu and Tawata [18], with minor modifications. Briefly, 20 μL of UP extract at a concentration of 1 mg/mL was added to a 96-well plate, followed by 200 μL of the N-Succinyl-Ala-Ala-Ala-Ala-Ala-Ala-p-nitroanilide elastase substrate in 0.1 M Tris-HCl buffer (pH 8.0). The mixture was incubated at 25 °C for 10 min. Afterward, 20 μL of elastase from porcine pancreas (0.03 units/mL) was added, mixed, and incubated again at 25 °C for 10 min. Absorbance was measured at 410 nm using a microplate reader (Eon, BioTek Instruments, Winooski, VT, USA). Ascorbic acid was used as the positive control. The inhibition activity was computed using the formula below.

Elastase inhibition assay (%) = [1 − (EA_s_/EA_c_)] × 100 (3)

where

EA_s_ = enzyme activity of the sample

EA_c_ = enzyme activity of the control

#### 2.3.4. Collagenase Inhibitory Assay

The collagenase inhibitory activity of sea mustard extracts was determined using the method of Chatatikum and Chiabchalard [19], with minor modifications. Briefly, 20 μL of UP extract at a concentration of 1 mg/mL was added to a 96-well plate, followed by 80 μL of collagenase from Clostridium histolyticum (0.25 units/mL) in 50 mM Tricine buffer containing 10 mM CaCl_2_ and 400 mM NaCl. The mixture was incubated at room temperature for 15 min. Subsequently, 100 μL of N-[3-(2-Furyl) acryloyl]-Leu-Gly-Pro-Ala was added and mixed. The solution was incubated again at room temperature for 20 min. Absorbance was measured at 410 nm using a microplate reader (Eon, BioTek Instruments, Winooski, VT, USA). Ascorbic acid was used as the positive control. The inhibition activity was computed using the formula below.

Collagenase inhibition assay (%) = [1 − (EA_s_/EA_c_)] × 100 (4)

where

EA_s_ = enzyme activity of the sample

EA_c_ = enzyme activity of the control

### 2.4. α-Glucosidase Inhibitory Assay

The α-glucosidase inhibition activity of UP extracts was determined using the method of Oboh et al. [20], with some modifications. Briefly, 20 μL of UP extracts at different concentrations (0.125, 0.25, 0.5, 1 mg/mL) were added in a 96-well plate. Then, 100 μL of α-glucosidase enzyme from S. cerevisiae (0.06 U/mL) in 0.1 M potassium phosphate buffer (pH 7.0) was added and reacted for 5 min at 25 °C. Afterwards, 100 μL of 5 mM p-Nitrophenyl-α-D-glucopyranoside in 0.1 M potassium phosphate buffer (pH 7.0) was added and reacted for 10 min at 25 °C. The absorbance was read at 405 nm using a microplate reader (Eon, BioTek, Instruments, Winooski, VT, USA). Acarbose was used as the positive control with concentrations from 125 μg/mL to 1000 μg/mL. The % inhibition was computed using the formula below.

α-Glucosidase inhibition (%) = [(A_c_ − A_s_)/A_c_] × 100 (5)

where

A_c_ = Absorbance of control

A_s_ = Absorbance of sample

### 2.5. Xanthine Oxidase Inhibitory Assay

The xanthine oxidase inhibition activity of UP extracts was determined using the method of Umamaheswari et al. [21]. Briefly, 1 mL of UP extracts was added with 2.9 mL of 0.1 M phosphate buffer (7.5 pH) and 0.1 mL xanthine oxidase from bovine milk solution (0.01 unit/mL in phosphate buffer). Then it was incubated at 25 °C for 15 min. Then, 2 mL of substrate solution (150 μM xanthine in phosphate buffer) was added and incubated 25 °C for 30 min. To stop the reaction, 1 mL of 1 N HCl was added, and absorbance was measured at 290 nm using a microplate reader (Eon, BioTek, Instruments, Winooski, VT, USA). Allopurinol was used as a positive control with concentrations from 125 μg/mL to 1000 μg/mL. The % inhibition was computed using the formula below.

Xanthine oxidase inhibition (%) = [(A_c_ − A_s_)/Ac] × 100 (6)

where

A_c_ = Absorbance of control

A_s_ = Absorbance of sample

### 2.6. Cell-Based Assays

#### 2.6.1. Cell Culture

The mouse fibroblast cell line (L929) was maintained in a humidified incubator at 37 °C with a 5% CO_2_ atmosphere. L929 cells were cultured in RPMI 1640 medium under normal conditions, while high-glucose conditions were achieved using high-glucose DMEM medium (Dulbecco’s Modified Eagle Medium) (Thermo Fisher Scientific (Waltham, MA, USA). Both media were supplemented with 10% fetal bovine serum and 1% antibiotic solution containing streptomycin (100 μg/mL) and penicillin (100 units/mL) [22].

**Figure 1:**
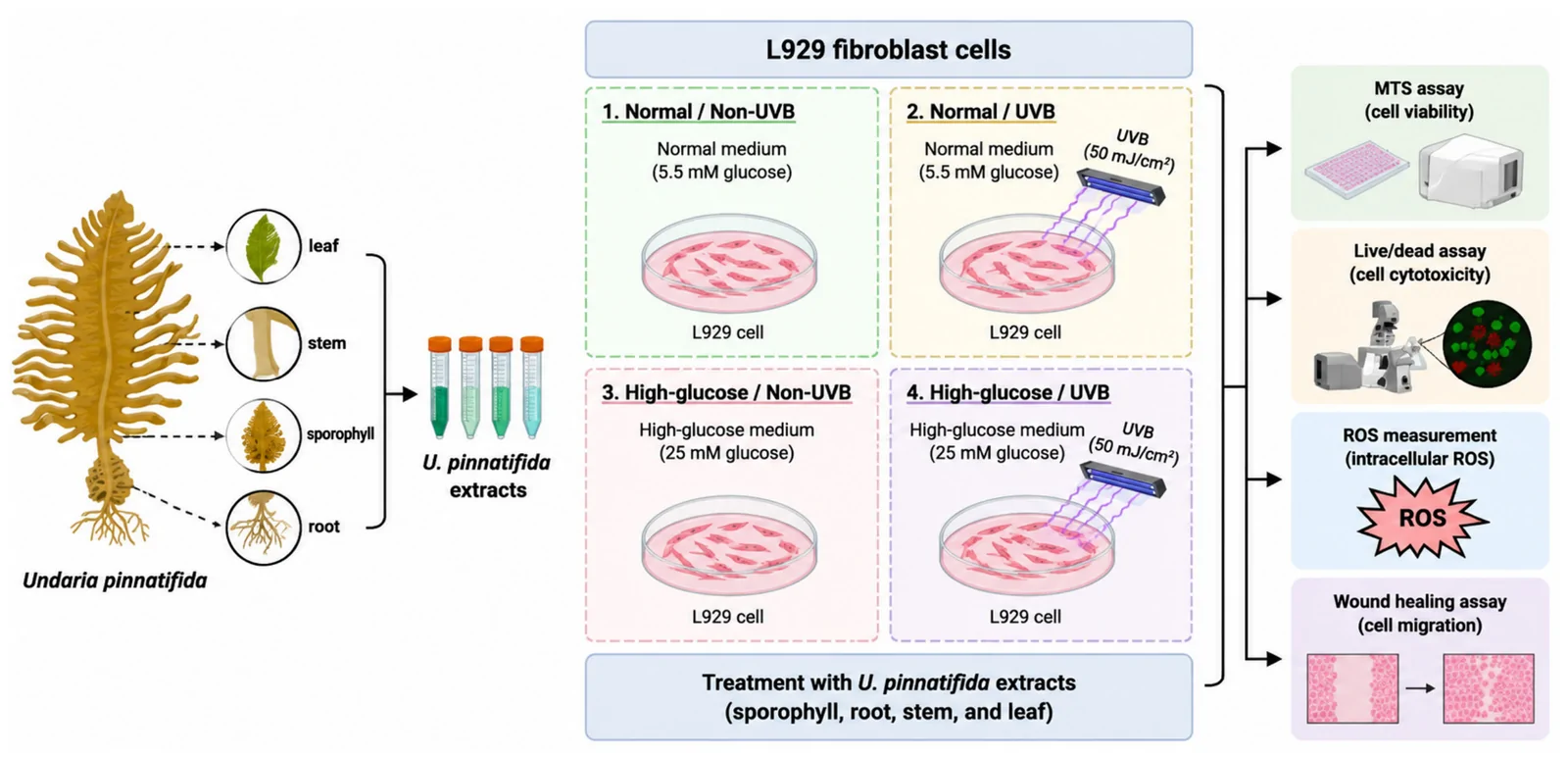
Schematic overview of the experimental design for evaluating *Undaria pinnatifida* extracts in L929 fibroblasts under four experimental conditions: (1) normal/non-UVB, (2) normal/UVB, (3) high-glucose/non-UVB, and (4) high-glucose/UVB.

The UVB exposure was carried out using a UVB Handy Lamp (U01-02-145, LK Lab, Namyangju, Republic of Korea) at a dose of 50 mJ/cm^2^, and the light intensity was measured with a UV light meter (Lutron 1907-UV340A, Taiwan, China). The experimental design comprised four conditions based on culture medium and UVB irradiation: normal/non-UVB, normal/UVB, high-glucose/non-UVB, and high-glucose/UVB, as illustrated in Figure 1.

#### 2.6.2. Cell Viability Assay (MTS Assay)

MTS assay was performed to assess cellular metabolic activity using the CellTiter 96^®^ AQueous One Solution Cell Proliferation Assay Kit (Promega Corp., Madison, WI, USA). Preliminary concentration-screening experiments were conducted prior to the comparative cell-based assays, and 50 μg/mL was selected as a concentration that produced a measurable cellular response and was suitable for comparative screening. This concentration was subsequently applied as a common screening concentration to enable consistent comparison among the different samples under the same experimental conditions. L929 cells were seeded into a 96-well plate at a concentration of 1 × 10^5^ cells/mL. After 48 h, 20 μL of UP extracts (50 μg/mL) was added to each well. For UVB-irradiated cells, UVB exposure was applied after the addition of the UP extracts. Following UVB irradiation, the cells were incubated at 37 °C in a humidified atmosphere with 5% CO_2_ for 24 h. Subsequently, 30 μL of the MTS reagent was added to each well, and the cells were incubated for 20 min under the same conditions. For non-irradiated cells, the corresponding extract-treated cells were incubated under the same culture conditions before addition of the MTS reagent. The optical density (OD) was measured at 490 nm using a microplate reader (Eon, BioTek Instruments, Winooski, VT, USA). Relative MTS response (%) for both non-irradiated and UVB-irradiated cells was calculated using the formula provided below.

Relative MTS response (%) = [(A_cs_ − A_b_)/(A_c_ − A_b_)] × 100 (7)

where

A_cs_ = Absorbance of samples-treated cells

A_b_ = Absorbance of blank

A_c_ = Absorbance of control

#### 2.6.3. Fluorescence Examination of Live/Dead Cells

The live/dead cell assay was performed to evaluate the toxicity of the samples on L929 cells using the R37601 LIVE/DEAD™ Cell Imaging Kit (Thermo Fisher Scientific, Waltham, MA, USA). Briefly, 1 × 10^5^ cells/mL were seeded in a 6-well plate and incubated for 24 h at 37 °C with 5% CO_2_. After incubation, the culture medium was replaced with fresh ones, and 50 μL of various UP extracts at a concentration of 50 μg/mL were added. The cells were then incubated for an additional 24 h. After the second incubation, 50 μL of the double-staining kit working solution (a mixture of 5 μL calcein AM, 20 μL ethidium homodimer-1, and 10 mL DPBS) was added, and the cells were incubated for 20 min under the same conditions. For UVB-irradiated cells, UVB exposure was applied after the addition of UP extracts. The cells were observed using an A1 HD25 Nikon confocal microscope (© 2025 Nikon Instruments Inc., Tokyo, Japan). Live cells were stained green, while dead cells were stained red. Quantification and analysis were performed using ImageJ 1.54g software [23].

#### 2.6.4. Measurement of Intracellular ROS Levels

Intracellular ROS levels were evaluated using two complementary approaches: a 96-well plate-based measurement using a microplate reader and DHE fluorescence imaging followed by image-based fluorescence quantification. The MAK142 Fluorometric Intracellular ROS kit (Sigma-Aldrich, St. Louis, MO, USA) was used to measure intracellular ROS levels. For the 96-well plate-based assay, cells were seeded at a concentration of 1.5–1.7 × 10^5^ cells/mL in a 96-well plate and cultured for 48 h. After discarding the medium, cells were resuspended in 100 μL of fresh medium, and 30 μL of various UP extracts (50 μg/mL) was added. A 50 μM master reaction mix, prepared by combining 20 μL of ROS detection reagent stock solution with 10 mL of assay buffer, was then added, followed by incubation at 37 °C with 5% CO_2_ for 30 min. For non-irradiated cells, ROS generation was induced by adding 3% H_2_O_2_ and incubating for another 30 min under the same conditions. For UVB-irradiated cells, the same procedure was followed up to the addition of the master reaction mix. The cells were then exposed to UVB irradiation and subsequently incubated at 37 °C with 5% CO_2_ for 1 h in the dark. For the plate-reader measurement, absorbance (optical density, OD) was measured at 525 nm using a microplate reader (Eon, BioTek Instruments, Winooski, VT, USA), and ROS generation (%) was calculated from the absorbance values using the following equation.

ROS generation (%) = [1 − ((A_c_ − A_s_)/A_c_)] × 100 (8)

where

A_c_ = Absorbance of control

A_s_ = Absorbance of sample

The same ROS kit was used to assess ROS levels via DHE fluorescence intensity. Briefly, L929 cells were seeded in 6-well plates at a concentration of 1.5–1.7 × 10^5^ cells/mL and cultured for 48 h. After removing the old medium, the cells were rinsed twice with PBS, followed by the addition of fresh medium, 50 μL of various UP extracts, and 50 μM of the master reaction mix. The cells were then incubated at 37 °C with 5% CO_2_ for 30 min. For non-irradiated cells, ROS levels were induced by adding 3% H_2_O_2_, and the cells were incubated for an additional 30 min under the same conditions.

For UVB-irradiated cells, the same procedure was followed up to the addition of the master reaction mix. The cells were then exposed to UVB radiation and subsequently incubated at 37 °C with 5% CO_2_ for 1 h in the dark. ROS fluorescence was visualized using an A1 HD25 Nikon confocal microscope (Nikon Instruments, Melville, NY, USA). DHE-derived red fluorescence was detected within the excitation range of 488–535 nm (optimal excitation approximately 518 nm), with a maximum emission at approximately 610 nm, according to the fluorescence characteristics of the probe. Fluorescence intensity (dark red) was analyzed using ImageJ software [24].

#### 2.6.5. In Vitro Scratch Wound-Closure Assay

The scratch assay was performed based on the method of Premarathna et al. [25], with some modifications. For non-irradiated cells, fibroblasts were seeded in a 6-well plate at a concentration of 1 × 10^5^ cells/mL and cultured until they reached 80% confluency. A small linear scratch was created using a sterile 200 μL pipette tip, and the cells were rinsed twice with PBS to remove any cellular debris. Then, 1 mL of fresh medium and 50 μL of UP extracts (50 μg/mL) were added and gently mixed. The cells were incubated at 37 °C with 5% CO_2_ for up to 48 h, and scratch closure was observed at 0, 12, 24, 36, and 48 h. Images were captured using an inverted microscope (Leica DMi1, Wetzlar, Germany), and scratch width was determined by measuring changes in the width of the cell-free scratched area using Nano Measurer 1.2 software. The percentage of scratch closure was calculated using the formula below.

Scratch closure (%) = [(W_0_ − W_t_)/W_0_] × 100 (9)

where

W_0_ = Initial scratch width at 0 h

W_t_ = Remaining scratch width at time t

For UVB-irradiated cells, the same procedure was followed, except that after the addition of UP extracts, the cells were exposed to UVB radiation at 50 mJ/cm^2^ before being incubated under the same conditions. After UVB exposure, cells were incubated at 37 °C with 5% CO_2_ for up to 48 h, and scratch closure was monitored at 0, 12, 24, 36, and 48 h. Changes in scratch width were measured, and scratch-closure percentages were calculated as described for the non-irradiated group.

### 2.7. Statistical Analysis

Statistical analyses were performed using SPSS version 22.0 (SPSS Inc., Chicago, IL, USA). Data are presented as the mean ± SD, with the number of replicates specified in the corresponding figure legends. Differences between two sample groups were assessed using an independent *t*-test, whereas one-way ANOVA followed by Duncan’s multiple range test was employed for comparisons among multiple groups. For the time-course scratch-closure assay, the effects of treatment, time, and the treatment × time interaction were evaluated using two-way repeated-measures ANOVA. Duncan’s multiple range test was used for comparisons among treatments at each time point and among time points within each treatment. Statistical significance was defined at *p* < 0.05.

## 3. Results and Discussion

### 3.1. Tyrosinase Inhibitory Activity

Tyrosinase plays a critical role in the complex process of melanogenesis [26]. While melanin provides photoprotective benefits, the excessive production of melanin in certain areas of the skin can lead to hyperpigmentation, which is often considered an aesthetic concern. Consequently, tyrosinase inhibitors are important components in skin-lightening agents and are widely incorporated into cosmetic formulations.

**Figure 2:**
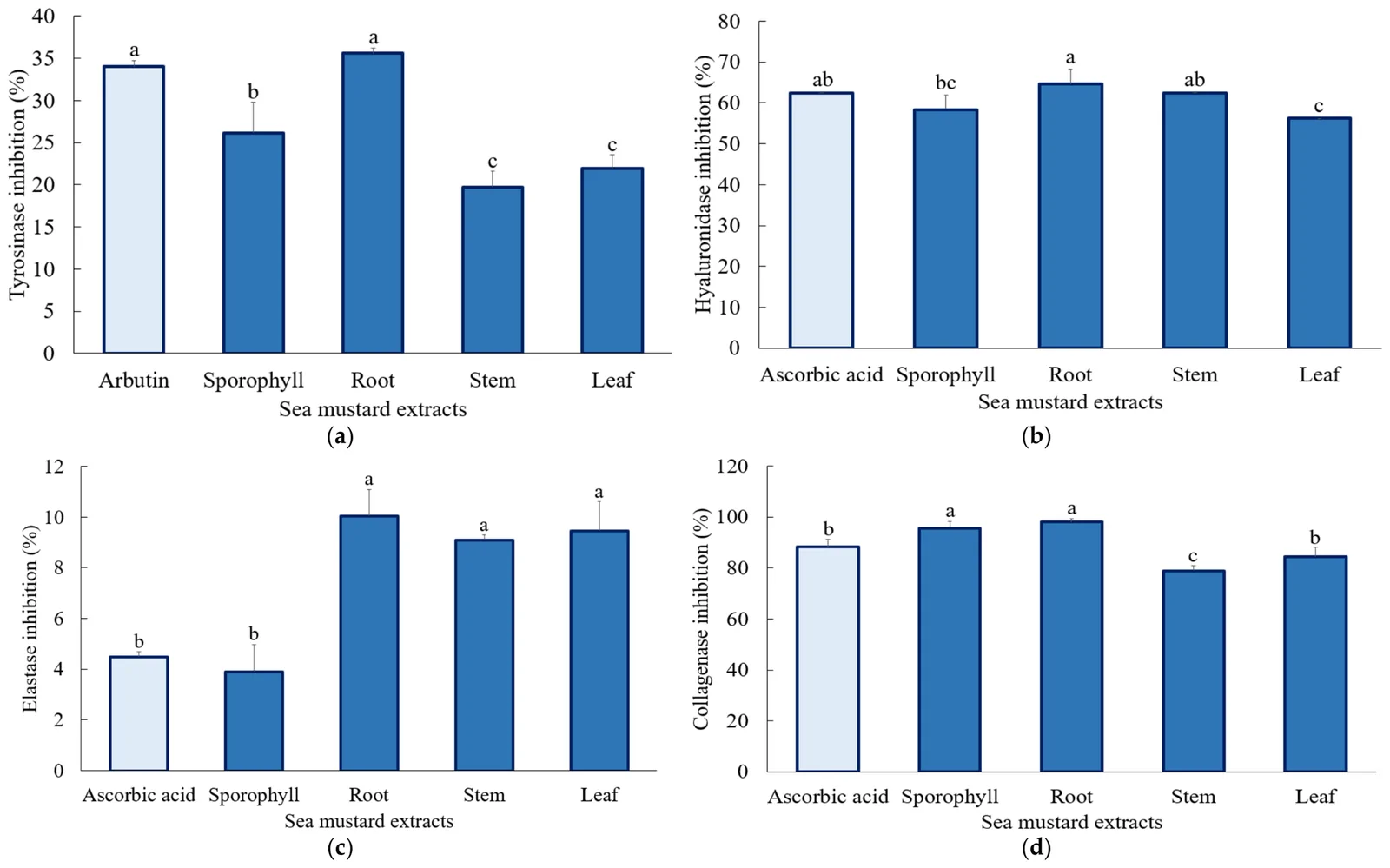
(**a**) Tyrosinase; (**b**) hyaluronidase; (**c**) elastase; (**d**) collagenase inhibitory activities (%) of *U. pinnatifida* extracts. Data are presented as the mean ± SD (n = 3). Different lowercase letters indicate significant differences among treatments according to one-way ANOVA followed by Duncan’s multiple range test (*p* < 0.05). Bars sharing the same letter are not significantly different.

The UP extracts exhibited tyrosinase inhibition with roots having the strongest inhibition among the samples (Figure 2a). The inhibitory activities of UP extracts ranged from 19.74% to 35.58%, with the root extract showing the strongest inhibition, followed by sporophyll, leaf, and stem. This effect may be related to bioactive constituents present in the root extract, including polyphenolic compounds; however, the chemical constituents responsible for the observed activity were not directly quantified in the present study. Polyphenolic compounds in brown seaweeds have been shown to effectively address hyperpigmentation and serve as promising agents in the development of skin-whitening products [27]. For instance, Jang et al. [28] demonstrated that 4-hydroxyphenethyl alcohol (4-HPEA), a phenolic compound isolated from the brown seaweed *Hizikia fusiformis*, exhibited depigmentation effects on UVB-induced hyperpigmented spots in guinea pig skin. Additionally, flavonoids, polysaccharides, and pigments from brown seaweeds have been reported to possess skin-whitening properties [29]. Moreover, findings of this study align with those of Lee et al. [30], who reported that the tyrosinase inhibitory activity of different *U. pinnatifida* parts (sporophyll, leaf, and stem) at a concentration of 1 mg/mL ranged from 27% to 36%.

### 3.2. Hyaluronidase, Elastase, and Collagenase Inhibitory Activities

Hyaluronidase, elastase, and collagenase are enzymes involved in the degradation of different components associated with skin connective tissues and the extracellular matrix (ECM) [31]. The skin enzyme hyaluronidase catalyzes the degradation of hyaluronic acid, a glycosaminoglycan with a remarkable ability to bind and retain water molecules, which plays a vital role in maintaining skin moisture and elasticity [32]. Elastase is a protease responsible for degrading elastin a key protein that provides elasticity to the skin, ligaments, and arteries. Collagenases degrade collagen, a major component of the extracellular matrix (ECM), which provides structural support and accounts for 80% of the skin’s dry weight, playing a critical role in its tensile strength [33].

Similar to its anti-tyrosinase activity, the root extract of sea mustard exhibited the highest hyaluronidase, elastase, and collagenase inhibitory activities (*p* < 0.05), indicating its comparatively strong inhibitory activity against these ECM-related degrading enzymes in the cell-free assays. In the inhibition against hyaluronidase, statistical analysis indicated no significant difference in inhibitory activity between the root extract, ascorbic acid (positive control), and the stem extract while ascorbic acid and sporophyll extracts also exhibited comparable inhibitory activities (Figure 2b). These findings suggest that, while the root possesses the strongest inhibitory effect, other UP extracts, particularly the stem and sporophyll extracts, may also offer valuable anti-hyaluronidase properties. Hyaluronidase inhibition across sea mustard waste parts ranged from 56.25% to 64.58%, in the order of root > stem > sporophyll > leaf. Recent studies have further highlighted the anti-hyaluronidase properties of other bioactive compounds in sea mustard, such as alginate, fucoidan, and phlorotannin. Arunkumar et al. [34] demonstrated that phlorotannin extracts from the stipe and blade of various brown seaweed species exhibited the highest anti-hyaluronidase activity, followed by fucoidan and alginate extracts.

As for elastase inhibitory activity, all UP extracts showed inhibition ranging from 3.90% to 10.05% at a concentration of 1 mg/mL. The highest inhibition was observed in the roots, with no significant difference compared to the stem and leaf extracts (Figure 2c). In contrast, the sporophyll showed lower elastase inhibition, with no significant difference compared to ascorbic acid (positive control). In the study by Lee et al. [30], water-extracted UP exhibited elastase inhibitory activity of 10.0 ± 0.2%, which is close to the values observed in this study.

The collagenase inhibitory activity of UP extracts ranged from 78.82% to 98.04% at 1 mg/mL, with the root extract showing the highest activity, followed by sporophyll, leaf, and stem (Figure 2d). Both root and sporophyll extracts exhibited significantly higher (*p* < 0.05) inhibition than ascorbic acid (positive control). Zárate et al. [35] reported collagenase inhibition above 50% in eight out of thirty seaweed species at 250 µg/mL, with one species reaching 81.7%.

It should be noted that the hyaluronidase, elastase, and collagenase inhibitory activities evaluated in the present study were determined using cell-free enzyme assays. Although inhibition of these enzymes can serve as an in vitro screening approach for identifying compounds with potential relevance to ECM degradation, these results do not demonstrate direct modulation of ECM remodeling in L929 fibroblasts. Cellular endpoints related to ECM homeostasis, including collagen synthesis or degradation, MMP expression, and hyaluronic acid metabolism, were not evaluated in the present study. Therefore, the observed enzyme inhibitory activities should be interpreted as preliminary in vitro screening results rather than direct evidence of anti-aging, anti-wrinkle, or cellular ECM-protective effects.

### 3.3. α-Glucosidase Inhibitory Activity

The inhibition of α-glucosidase can delay carbohydrate hydrolysis and is therefore widely used as an in vitro screening endpoint for compounds potentially affecting postprandial carbohydrate digestion. In the present study, the α-glucosidase inhibitory activity of UP extracts was compared based on their IC_50_ values. Among the samples, the root extract exhibited the lowest IC_50_ value, indicating the highest inhibitory activity, with an IC_50_ six times lower than the positive control, acarbose, while the sporophyll extract showed an approximately three-fold lower value (Table 1). Marine macroalgae contain various bioactive compounds, including phlorotannins, fucoxanthin, fucoidan, and polysaccharides, some of which have been associated with glucose-related biological activities [36,37]. However, the present α-glucosidase assay represents a cell-free in vitro screening system, and these findings should not be interpreted as evidence of antidiabetic efficacy or blood glucose regulation in vivo. Further studies using appropriate animal models and ultimately clinical investigations are required to establish the physiological relevance of these inhibitory activities.

### 3.4. Xanthine Oxidase Inhibitory Activity

The enzyme xanthine oxidase (XO) facilitates the oxidation of xanthine and hypoxanthine to uric acid in the human body, primarily produced in the liver and intestine. During this oxidation process, it generates superoxide, which can contribute to inflammatory diseases, vascular injuries, and chronic heart failure [38]. While synthetic drugs like allopurinol are effective XO inhibitors, prolonged use can cause serious side effects, including skin allergies, Stevens–Johnson syndrome, migraines, and nephropathy [21]. Currently, there is significant interest in finding natural XO inhibitors with fewer side effects.

Among the sea mustard extracts, the root exhibited the lowest IC50 value at 0.65 μg/mL (Table 1). Moreover, both root and stem extracts showed strong IC50 values lower than 1 μg/mL. Same with the results of the α-glucosidase inhibition assay, sea mustard waste parts also performed better than the commonly consumed leaf extract. In studies by Sarithakumari & Kurup [39] and Rafiquzzaman et al. [40], compounds like alginic acid and glycoprotein from seaweed demonstrated effectiveness in reducing inflammatory markers and inhibiting XO activity while enhancing antioxidant activity, suggesting their potential use in managing inflammatory and oxidative stress-related diseases.

### 3.5. Cell-Based Assay

#### 3.5.1. MTS Assay

**Figure 3:**
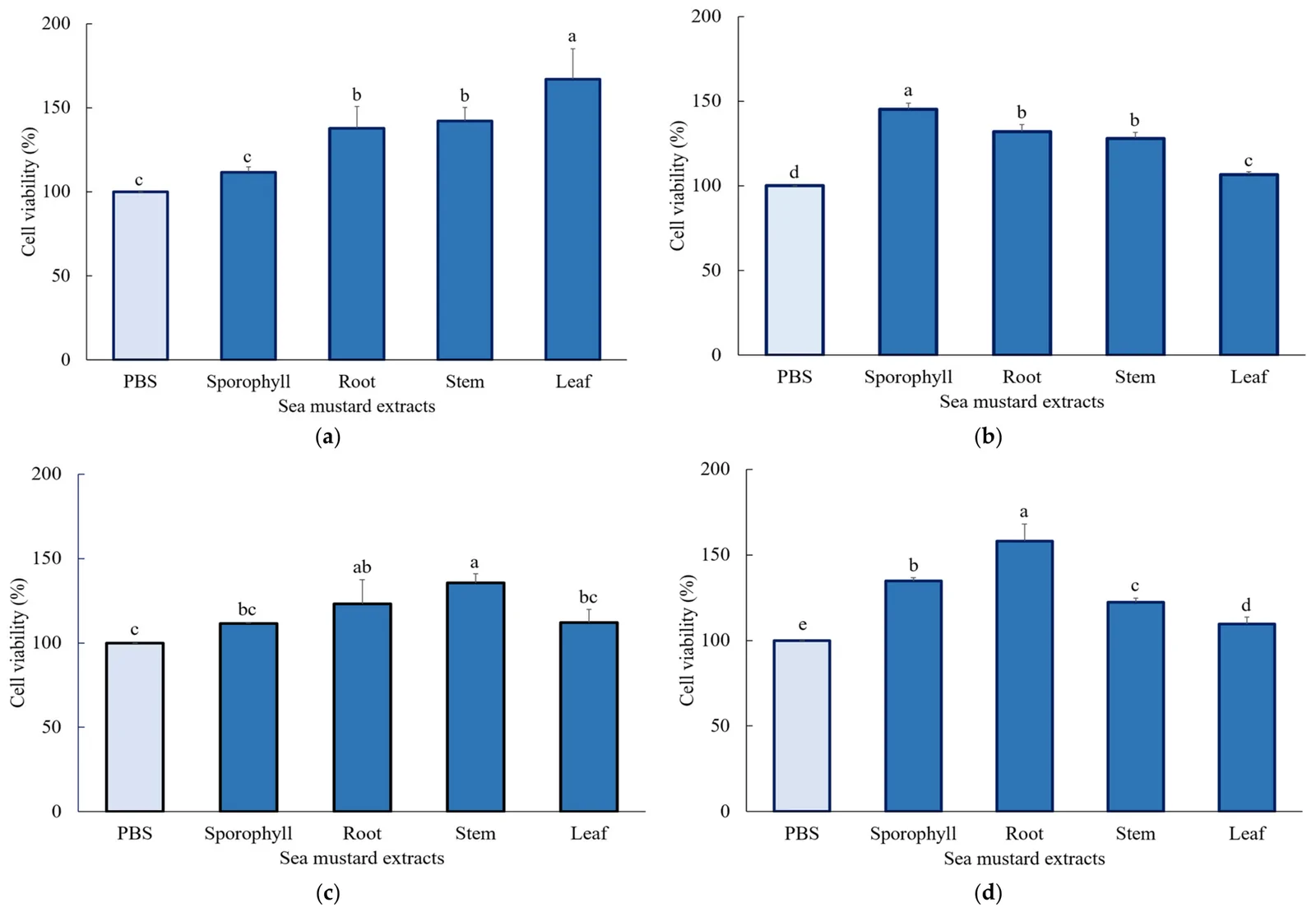
(**a**) Relative MTS response (% of control) in non-irradiated L929 cells under the normal culture condition; (**b**) UVB-irradiated L929 cells under the normal culture condition; (**c**) non-irradiated L929 cells under the high-glucose culture condition; and (**d**) UVB-irradiated L929 cells under the high-glucose culture condition treated with different *U. pinnatifida* extracts. Data are presented as the mean ± SD (n = 3). Different lowercase letters indicate significant differences among treatments according to one-way ANOVA followed by Duncan’s multiple range test (*p* < 0.05).

The effects of UP sporophyll, root, stem, and leaf extracts on the relative metabolic activity of L929 cells were assessed using the MTS assay. Under normal culture conditions, treatment with all UP extracts at 50 μg/mL resulted in relative MTS responses above 100% of the untreated control and significantly higher responses than the PBS-treated group. In non-irradiated cells (Figure 3a), relative MTS responses ranged from 111.52% to 166.87%, with the leaf extract showing the highest response, followed by the stem, root, and sporophyll extracts. Following UVB irradiation (Figure 3b), the sporophyll extract showed the highest relative MTS response, followed by the stem, root, and leaf extracts. Although the responses were generally lower after UVB irradiation than in non-irradiated cells, they remained above 100% of the untreated control, ranging from 106.63% to 145.31%.

Under high-glucose culture conditions, relative MTS responses also remained above 100% of the untreated control for all UP extract treatments. In non-irradiated cells (Figure 3c), the responses ranged from 111.59% to 135.44%, with the stem extract showing the highest response. In UVB-irradiated cells cultured under high-glucose conditions (Figure 3d), relative MTS responses ranged from 109.72% to 158.13%, with root extract being the highest. These results indicate that treatment with UP extracts at 50 μg/mL did not reduce the metabolic activity of L929 cells relative to the corresponding untreated controls under either normal- or high-glucose condition, with or without UVB irradiation. Rather, MTS responses remained above those of the untreated controls across all experimental conditions.

The MTS assay is based on the reduction in a tetrazolium compound to a colored formazan product by metabolically active cells and therefore primarily reflects cellular metabolic activity rather than cell number or proliferation directly [41]. Accordingly, MTS responses exceeding 100% of the untreated control should not necessarily be interpreted as enhanced cell viability or proliferation. Tetrazolium-based assays may be influenced by treatment-induced changes in cellular metabolism, and redox-active compounds present in natural extracts can potentially interfere with tetrazolium reduction, leading to over- or underestimation of cell viability [41,42]. Thus, the MTS results in the present study were interpreted together with the independent live/dead staining assay rather than as direct evidence of increased cell proliferation.

Previous studies nevertheless support the generally low cytotoxicity of brown algal materials, including *U. pinnatifida.* Phull et al. [43] reported no apparent toxicity of fucoidan isolated from *U. pinnatifida* in rats at doses up to 1000 mg/kg, while Guinea et al. [44] reported low toxicity of extracts from several commercial macroalgae, including *U. pinnatifida*, in UVB-exposed zebrafish embryos. Together with the live/dead staining results presented below, the MTS responses observed in the present study suggest that the UP extracts at the tested concentration did not exert marked cytotoxic effects on L929 cells. However, these findings should be interpreted within the limitations of the individual assays and should not be considered direct evidence of enhanced cell proliferation.

#### 3.5.2. Fluorescence Examination of Live/Dead Cells

**Figure 4:**
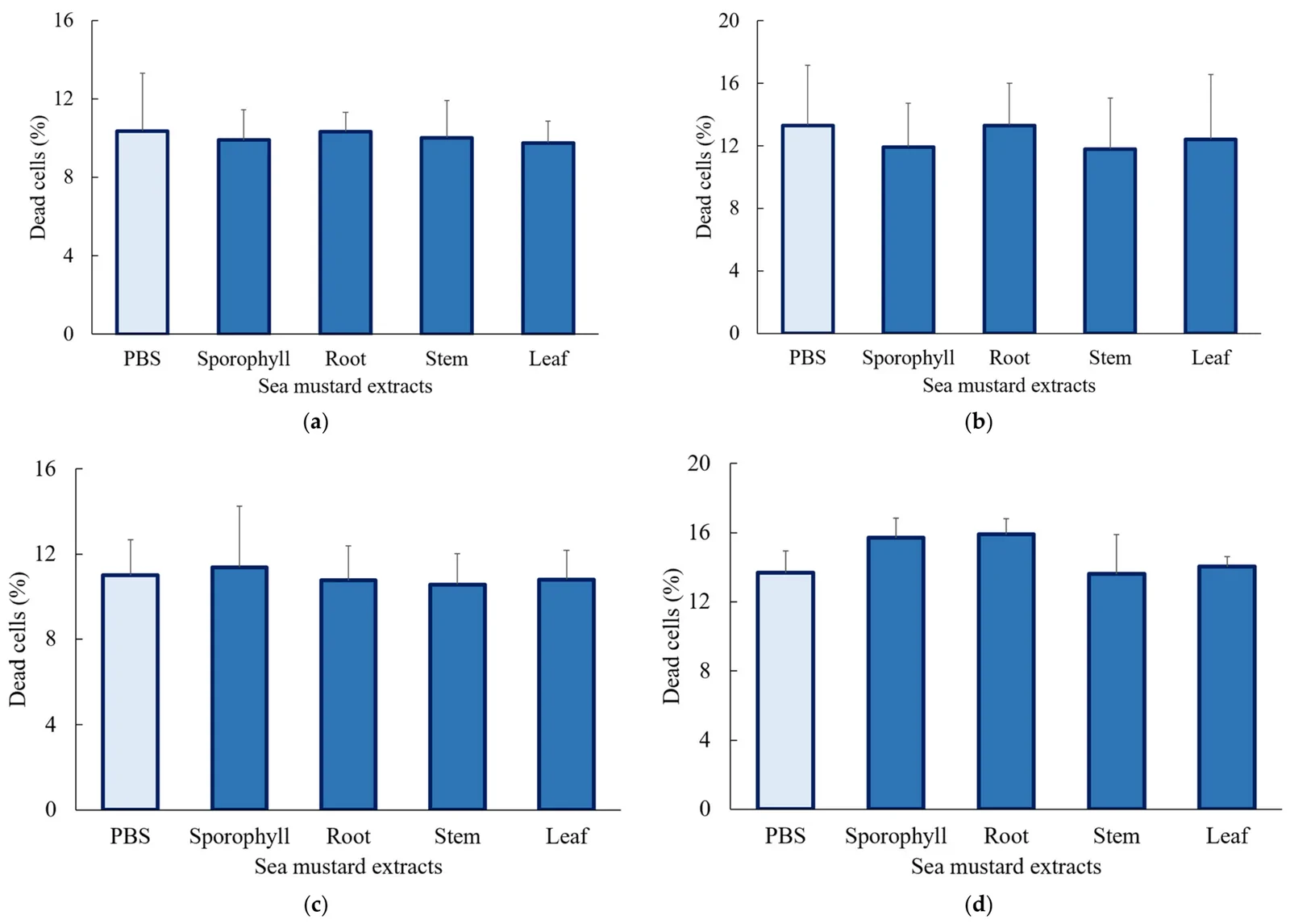
(**a**) Percentage of dead cells in non-irradiated L929 cells under the normal culture condition; (**b**) UVB-irradiated L929 cells under the normal culture condition; (**c**) non-irradiated L929 cells under the high-glucose culture condition; and (**d**) UVB-irradiated L929 cells under the high-glucose culture condition treated with different *U. pinnatifida* extracts. Data are presented as the mean ± SD (n = 3). Statistical differences among treatments were analyzed using one-way ANOVA followed by Duncan’s multiple range test. No significant differences were observed among treatments shown in (**a**–**d**).

**Figure 5:**
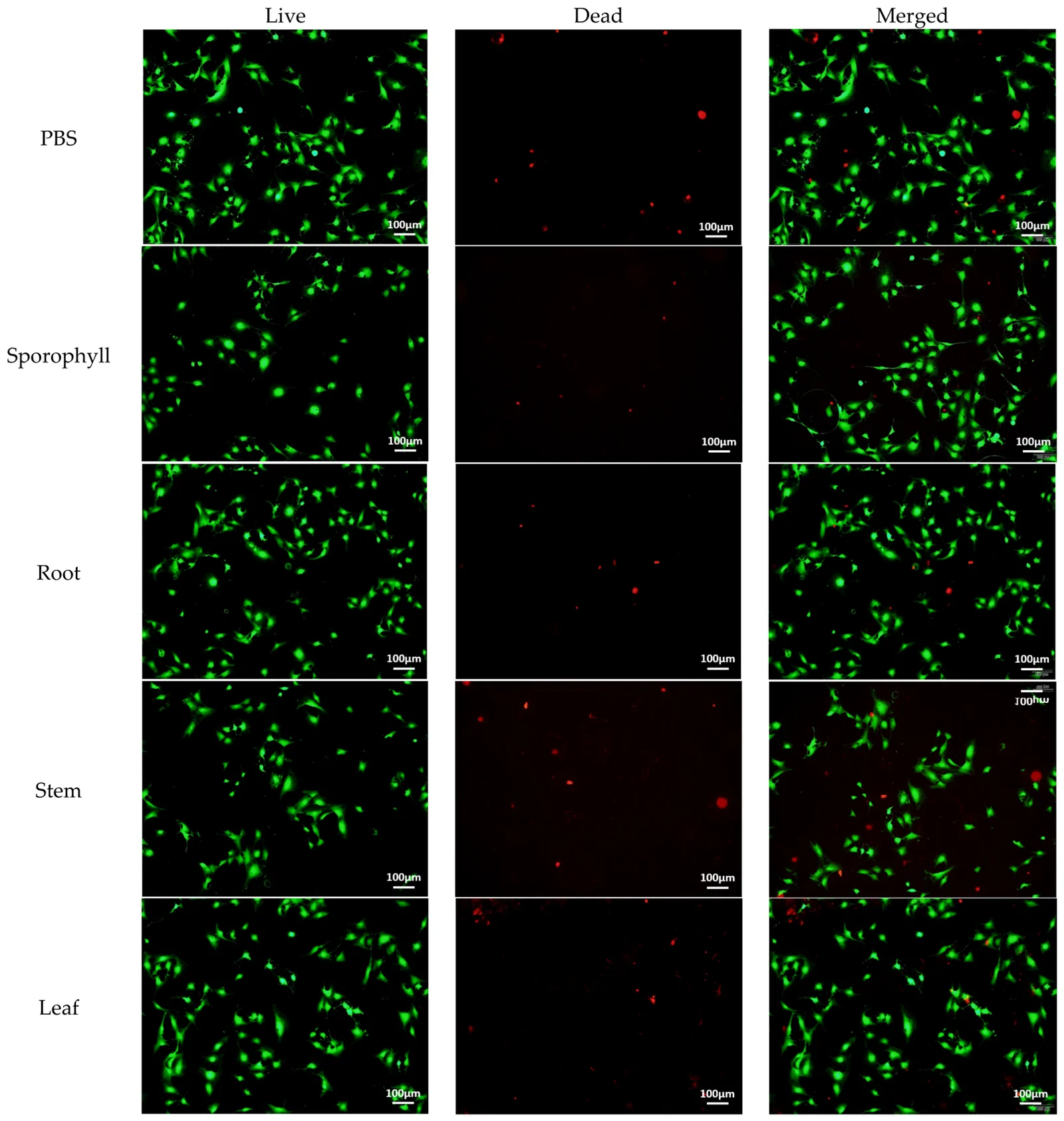
Live and dead staining of UVB-irradiated L929 cells under normal conditions treated with different *U. pinnatifida* extracts. Green fluorescence indicates live cells, while red fluorescence indicates dead cells.

The live/dead assay in non-irradiated L929 cells cultured under normal conditions demonstrated a minimal percentage of dead cells when treated with UP extracts, ranging from 9.75% to 10.33% (Figure 4a). Furthermore, no significant differences were observed among the treatments, including the control (PBS), indicating that the addition of any UP extract did not adversely affect the viability of L929 cells. As for UVB-irradiated cells, a slightly higher percentage of dead cells was observed compared to non-irradiated cells, which can be attributed to oxidative stress caused by UV radiation. The percentage of dead cells ranged from 11.81% to 13.31% in treatments with UP extracts, but no significant differences were also detected among the treatments (Figure 4b). As presented in Figure 5 and Figure S1, the majority of cells cultured under normal conditions were dyed green (live), with only a few dyed red (dead).

The percentage of dead cells under the high-glucose culture condition ranged from 10.56% to 11.38%, with no significant differences among the treatments (Figure 4c). A slightly higher percentage of dead cells was observed under the high-glucose culture condition than under the normal culture condition. However, because different basal media were used for these two culture conditions, this difference cannot be attributed exclusively to glucose concentration. Zhou et al. [45] reported reduced cell viability and proliferation in RSC96 cells cultured in high-glucose media compared to normal glucose conditions.

**Figure 6:**
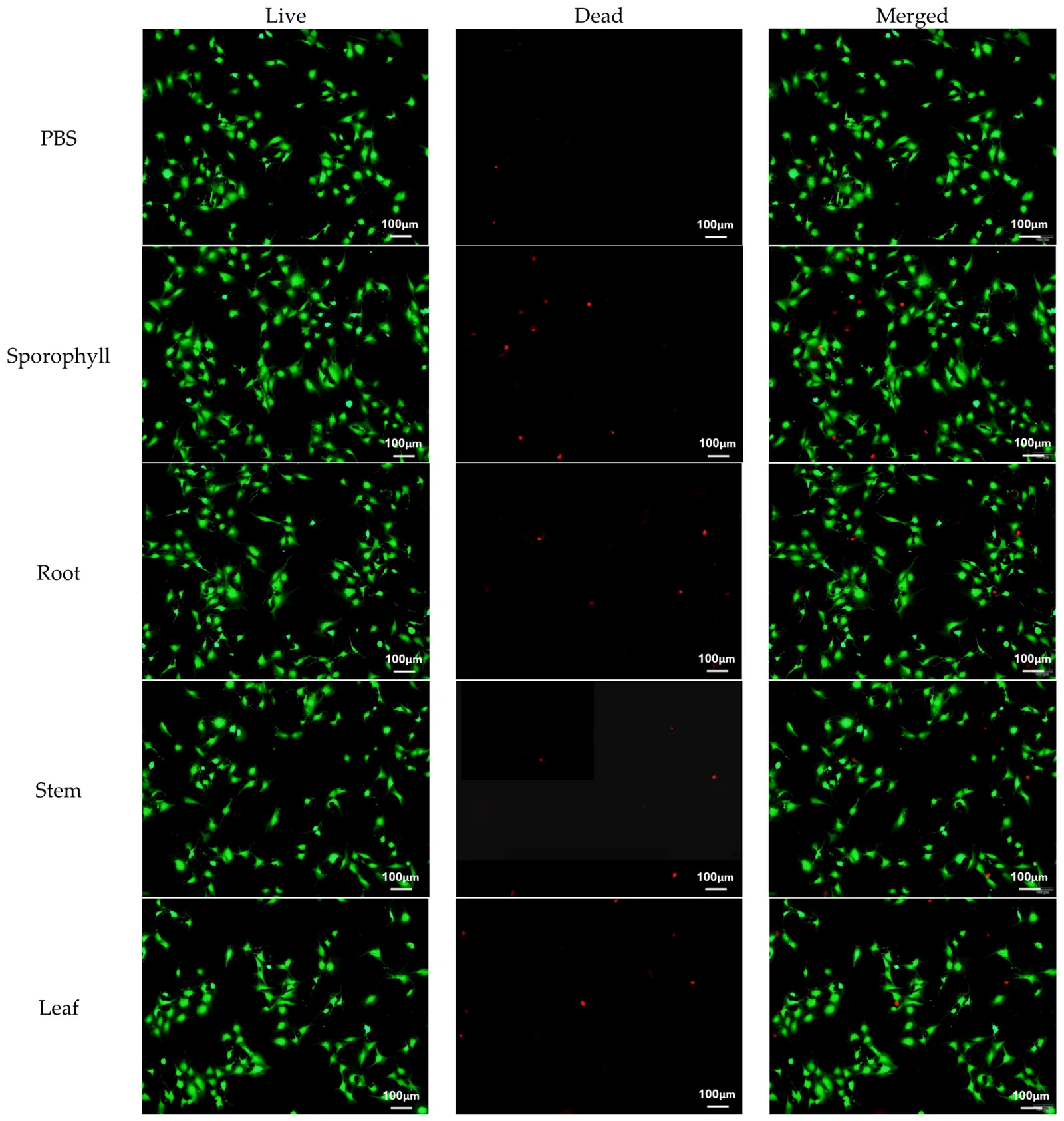
Live and dead staining of non-irradiated L929 cells under hyperglycemic conditions treated with different *U. pinnatifida* extracts. Green fluorescence indicates live cells, while red fluorescence indicates dead cells.

**Figure 7:**
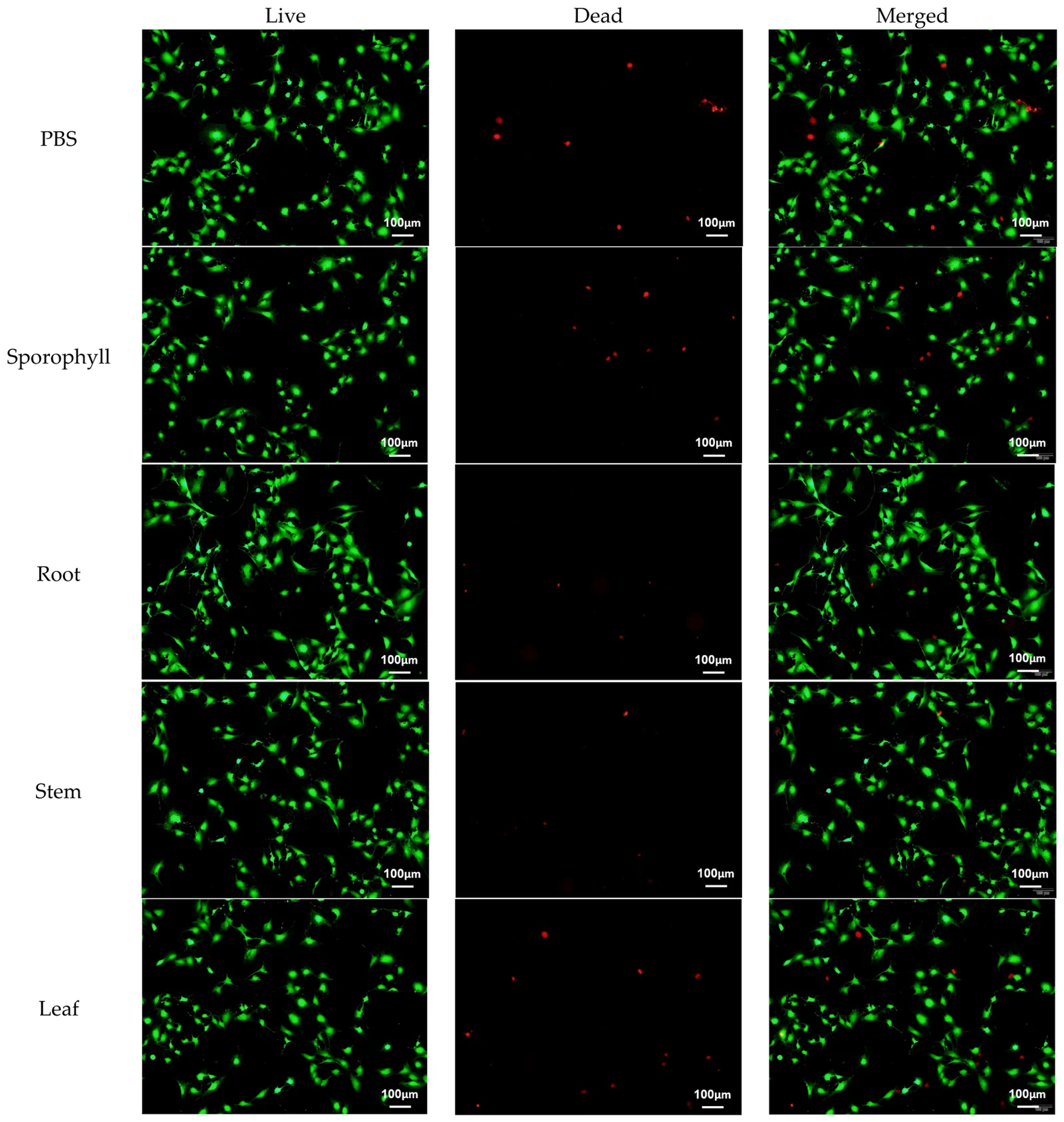
Live and dead staining of UVB-irradiated L929 cells under hyperglycemic conditions treated with different *U. pinnatifida* extracts. Green fluorescence indicates live cells, while red fluorescence indicates dead cells.

A higher percentage of dead cells was observed in UVB-irradiated L929 cells cultured under the high-glucose condition compared to non-irradiated cells. Previous studies have reported that high-glucose environments may increase cellular susceptibility to UVB-induced damage [28]. In the present study, however, the high-glucose condition was established using a different basal medium from that used for the normal culture condition; therefore, the observed differences should be interpreted within the specific culture conditions used rather than as glucose-specific effects. The percentage of dead cells following UVB exposure ranged from 13.61% to 15.89% in L929 cells cultured under the high-glucose condition and treated with UP extracts, showing no significant differences among the treatments (Figure 4d). Figure 6 and Figure 7 show representative live/dead staining images of non-irradiated and UVB-irradiated L929 cells, respectively, under the high-glucose culture condition.

Natural products such as seaweeds are known for their antioxidant properties, which contribute to their safety and non-toxic nature. Carvalho et al. [46] reported predominantly live L929 cells in cryogels containing fucoidan from the brown algae *Fucus vesiculosus*, highlighting its non-toxic nature. In addition, Silva et al. [47] observed that fucoidans extracted from *U. pinnatifida*, *F. vesiculosus*, and *Macrocystis pyrifera* significantly reduced H_2_O_2_-induced damage in RAW 264.7 macrophages, as evidenced by live/dead cell assays. These findings collectively support the biocompatibility of brown seaweeds in various cell lines.

#### 3.5.3. Intracellular ROS Scavenging Activity

The antioxidant properties of the UP extracts were assessed by measuring reactive oxygen species (ROS). ROS are a group of active molecules that play a crucial role in cell signaling and the cellular response to stress [48]. However, environmental factors or stressors, such as UV radiation, can cause an excessive increase in ROS levels, disrupting cellular homeostasis and potentially leading to cell death or apoptosis.

**Figure 8:**
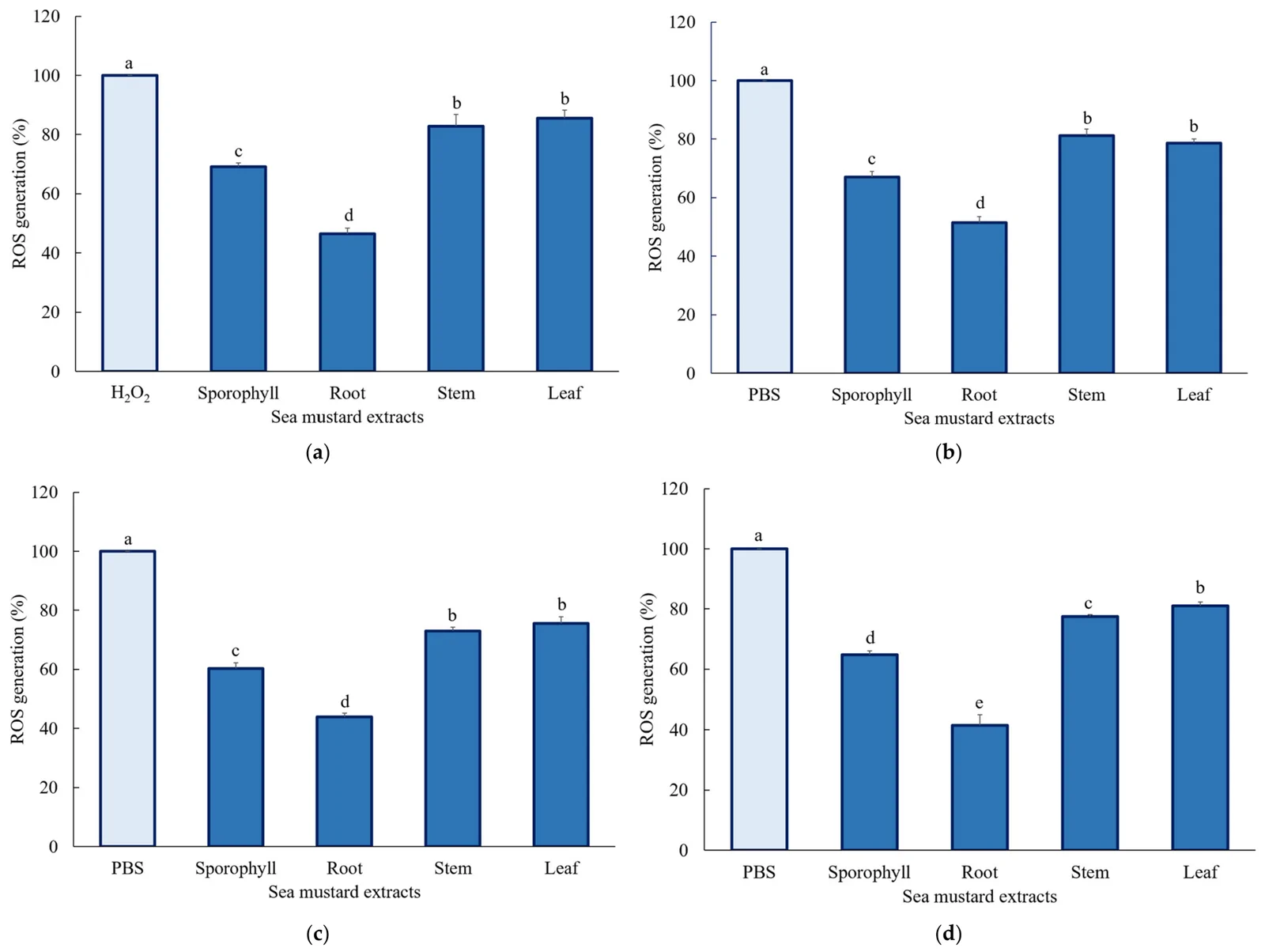
Intracellular ROS generation in (**a**) non-irradiated L929 cells under the normal culture condition; (**b**) UVB-irradiated L929 cells under the normal culture condition; (**c**) non-irradiated L929 cells under the high-glucose culture condition; and (**d**) UVB-irradiated L929 cells under the high-glucose culture condition treated with different *U pinnatifida* extracts. Data are presented as the mean ± SD (n = 3). Different lowercase letters indicate significant differences among treatments within each experimental condition according to one-way ANOVA followed by Duncan’s multiple range test (*p* < 0.05).

**Figure 9:**
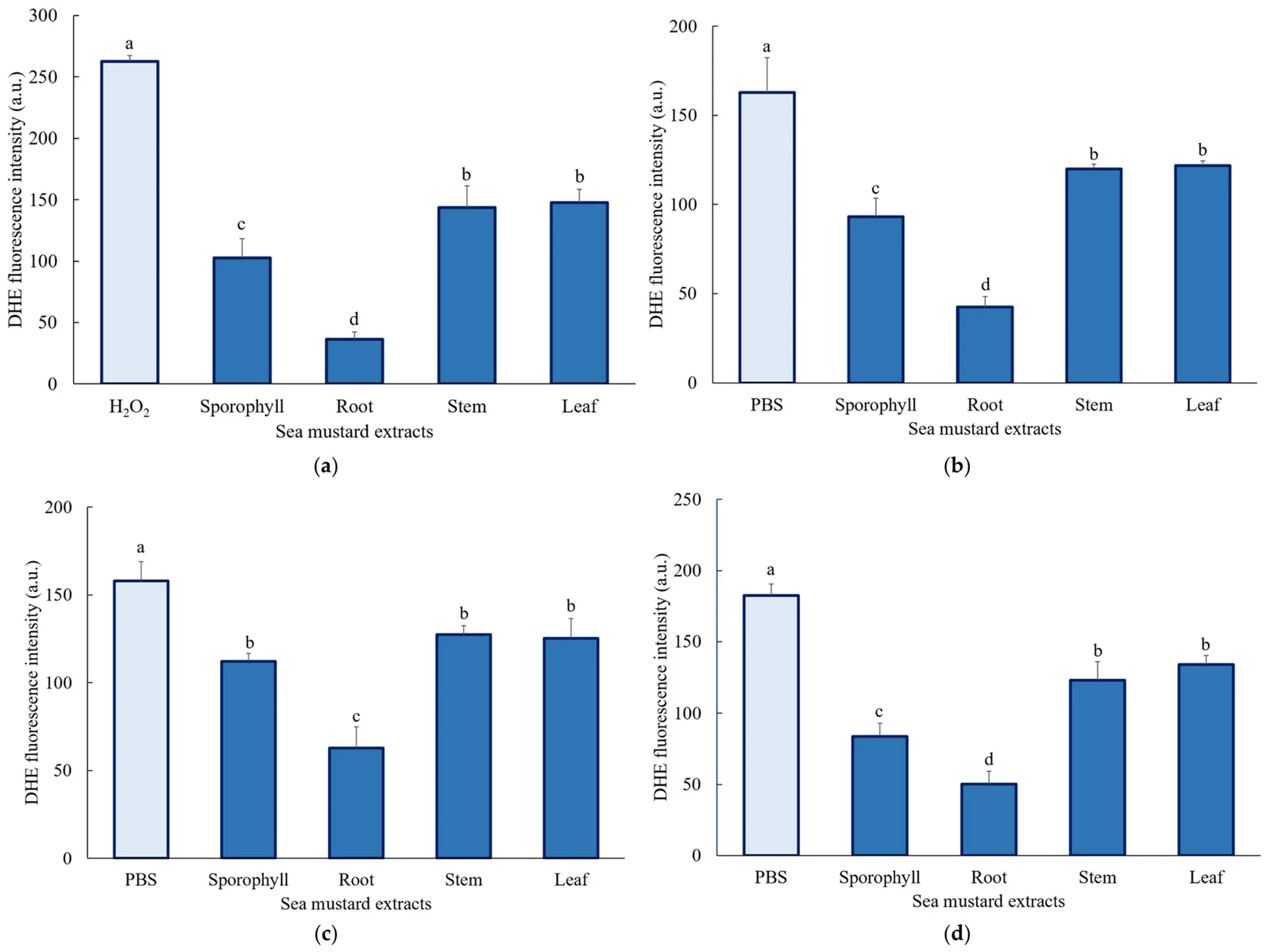
Dihydroethidium (DHE) fluorescence intensity (%) in (**a**) non-irradiated L929 cells under the normal culture condition; (**b**) UVB-irradiated L929 cells under the normal culture condition; (**c**) non-irradiated L929 cells under the high-glucose culture condition; and (**d**) UVB-irradiated L929 cells under the high-glucose culture condition treated with different *U. pinnatifida* extracts. Data are presented as the mean ± SD (n = 3). Different lowercase letters indicate significant differences among treatments within each experimental condition according to one-way ANOVA followed by Duncan’s multiple range test (*p* < 0.05).

To induce ROS production in non-irradiated normal L929 cells, H_2_O_2_ was introduced. All treatments with UP extracts resulted in significantly lower ROS levels (*p* < 0.05) compared to the untreated group. Among the treatments, the root extract demonstrated the highest reduction in ROS levels (53.56%), followed by sporophyll (30.89%), stem (17.11%), and leaf extracts (14.44%) (Figure 8a). These findings were consistent with the results of the ROS-specific fluorescent dye dihydroethidium (DHE) intensity measurements, where treatments with UP extracts significantly reduced (*p* < 0.05) fluorescence intensity compared to the untreated group (Figure 9a). DHE is oxidized by superoxide (O_2_˙^−^) within cells to produce 2-hydroxyethidium (2-OH-E+), which emits red fluorescence [49]. The root extract exhibited the lowest red fluorescence intensity (36.16 au), followed by sporophyll (102.85 au), stem (143.72 au), and leaf extracts (147.67 au). The results of this study are consistent with those reported by Kim et al. [50], which demonstrated that *Sargassum horneri* and *Dictyopteris divaricata*, both brown algae, exhibited significant ROS scavenging activity in RAW 264.7 cells at a concentration of 50 μg/mL. These findings indicate that UP extracts can effectively mitigate H_2_O_2_-induced oxidative stress in L929 cells.

UVB exposure induces photodamage in the skin by decreasing procollagen levels, which contributes to wrinkle formation, skin dryness, increased thickness, and undesired pigmentation. Additionally, UVB radiation elevates ROS generation, leading to oxidative damage to dermal proteins, which results in connective tissue disorganization and inflammation [51]. As shown in Figure 8b, UVB-irradiated L929 cells cultured under normal conditions exhibited significantly reduced ROS levels following the addition of various UP extracts compared to the untreated group. The root extract demonstrated the most substantial reduction in ROS levels (48.49%), followed by sporophyll (32.97%), leaf (21.34%), and stem (18.75%). Moreover, UP-treated cells had significantly lower (*p* < 0.05) DHE fluorescence intensity than the untreated cells, indicating reduced ROS levels (Figure 9b). The root extract exhibited the lowest fluorescence intensity (42.48 au), aligning with its highest ROS reduction potential. However, because the chemical composition of the extracts used in the present study was not quantitatively characterized, the observed cellular effects cannot be attributed to specific compounds. This outcome can be attributed to the high total polyphenol content and strong DPPH and ABTS radical scavenging activities of UP root, demonstrating its potent antioxidant properties. Similarly, lower DHE fluorescence intensities were observed with sporophyll (93.13 au), stem (120.05 au), and leaf (121.82 au) extracts compared to the untreated group, further supporting their ability to reduce intracellular ROS accumulation in UVB-irradiated cells. These findings are consistent with the study by Ji et al. [52], which reported that polysaccharides from *Sargassum fusiforme* reduced ROS production by 25% in UVB-irradiated HaCaT cells. Similarly, Ko et al. [53] found that extracts from *Ecklonia cava*, another brown alga, reduced ROS accumulation in HaCaT cells by approximately 40% after UV radiation exposure. The decreased ROS levels observed in UP-treated cells in the present study indicate an attenuation of intracellular ROS accumulation following UVB exposure. However, direct markers of UVB-induced DNA damage, such as CPDs or γ-H2AX, were not evaluated. Therefore, the observed reduction in intracellular ROS should not be interpreted as direct evidence that the UP extracts protected cells against UVB-induced DNA damage. Further studies incorporating specific DNA-damage endpoints are required to determine whether the extracts exert direct genomic photoprotective effects.

**Figure 10:**
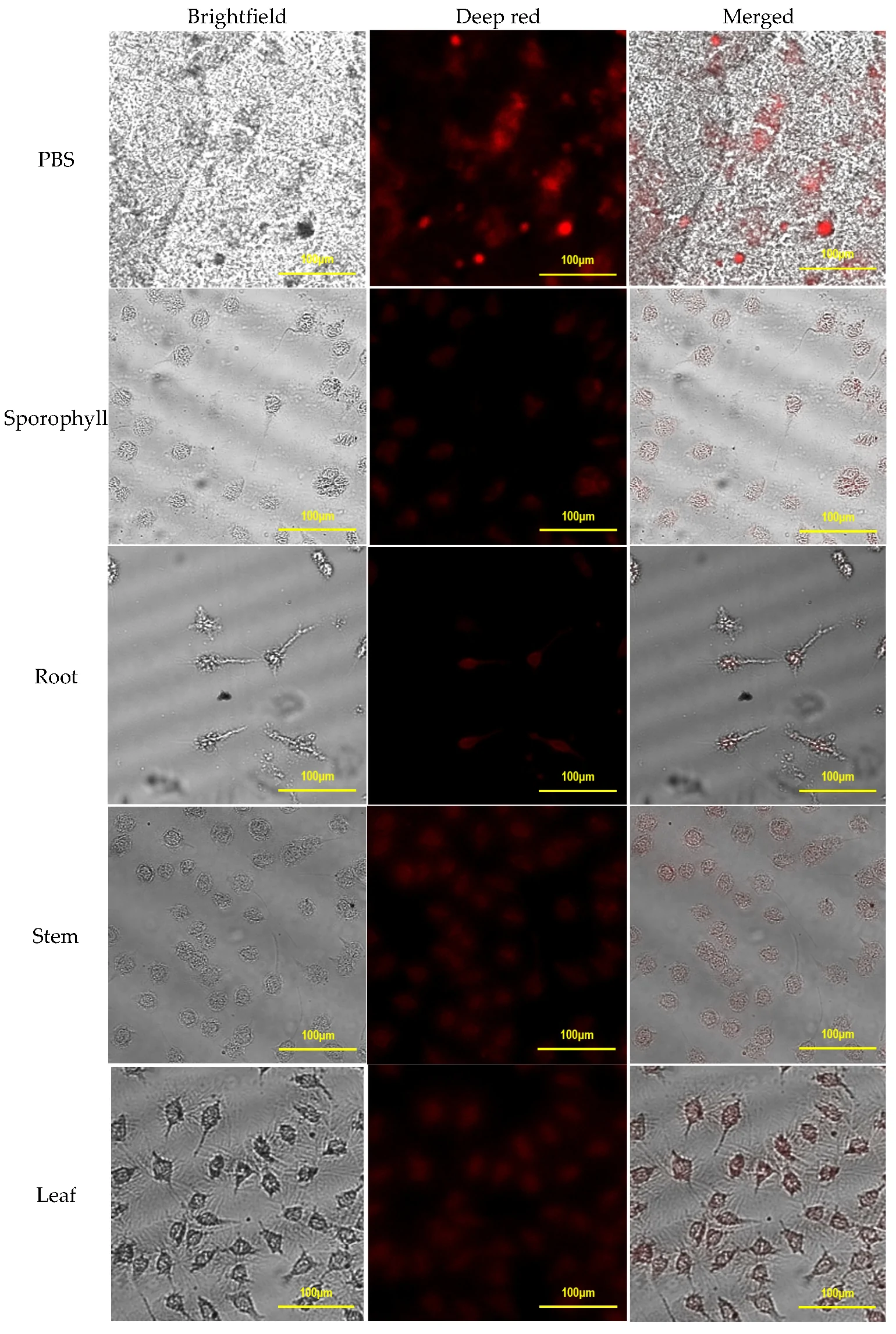
ROS generation in UVB-irradiated L929 cells under normal conditions treated with different *U. pinnatifida* extracts. Scale bars = 100 μm.

A high-glucose condition is known to induce elevated ROS levels, which can lead to mitochondrial dysfunction, inflammatory responses, and even apoptosis [54]. Previous studies have shown that prolonged exposure to high-glucose environments can alter cellular oxidative status and antioxidant defenses. As shown in Figure 8c, L929 cells cultured under the high-glucose condition and treated with UP extracts exhibited significant reductions in ROS levels. Consistent with the results of cells cultured under normal conditions, the root extract achieved the highest reduction at 56.13%, followed by sporophyll (39.64%), stem (26.93%), and leaf (24.36%). Figure 10 further corroborates these findings, illustrating weaker red fluorescence in cells treated with UP extracts, as verified by fluorescence intensity measurements, where all treatments with UP extracts exhibited significantly lower (*p* < 0.05) values compared to the untreated group. Among these, the root extract showed the lowest fluorescence intensity at 62.84 au, followed by sporophyll (112.20 au), leaf (125.38 au), and stem (127.33 au). In the study of De La Fuente et al. [55], brown algae *Cystoseira amentacea* extracts reduced ROS levels in L929 and RAW264.7 cells cultured in a high-glucose environment. In the present study, UP extracts also reduced ROS levels under the high-glucose culture condition used. However, because the normal and high-glucose conditions were established using different basal media, direct attribution of differences between these conditions to glucose concentration alone is not possible.

Reduced ROS levels were also observed in UVB-irradiated L929 cells cultured under the high-glucose condition when treated with UP extracts. Among the treatments, the root extract consistently demonstrated the most significant reduction in ROS generation, with a 58.48% decrease compared to the untreated group (Figure 8d). Other UP extracts also reduced ROS levels, with sporophyll achieving a 35.20% reduction, followed by stem (22.56%) and leaf (18.97%). Lowest fluorescence intensity was also observed in the root extract (50.31 au), followed by sporophyll (83.58 au), stem (122.72 au), and leaf (133.98 au) (Figure 9d). These results indicate that UP extracts reduced ROS levels in UVB-irradiated L929 cells under the high-glucose culture condition used in this study. Similar findings were reported by Ji et al. [52], where polysaccharides from *S. fusiforme* exhibited cytoprotective activity, particularly in terms of ROS reduction, against UVB-induced oxidative stress in HaCaT cells cultured in high-glucose environments.

**Figure 11:**
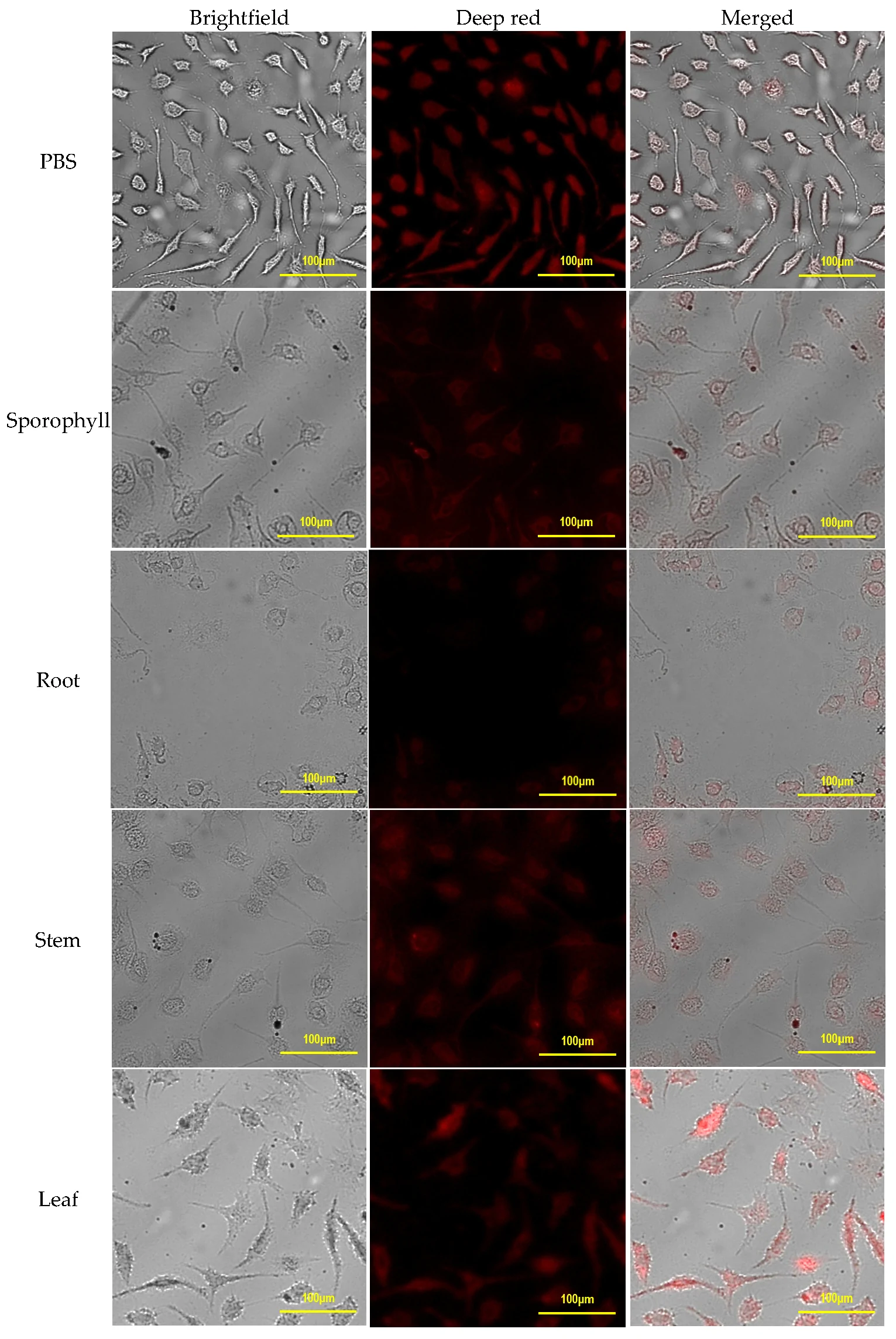
ROS generation in non-irradiated L929 cells under the high-glucose culture conditions treated with different *U. pinnatifida* extracts. Scale bars = 100 μm.

**Figure 12:**
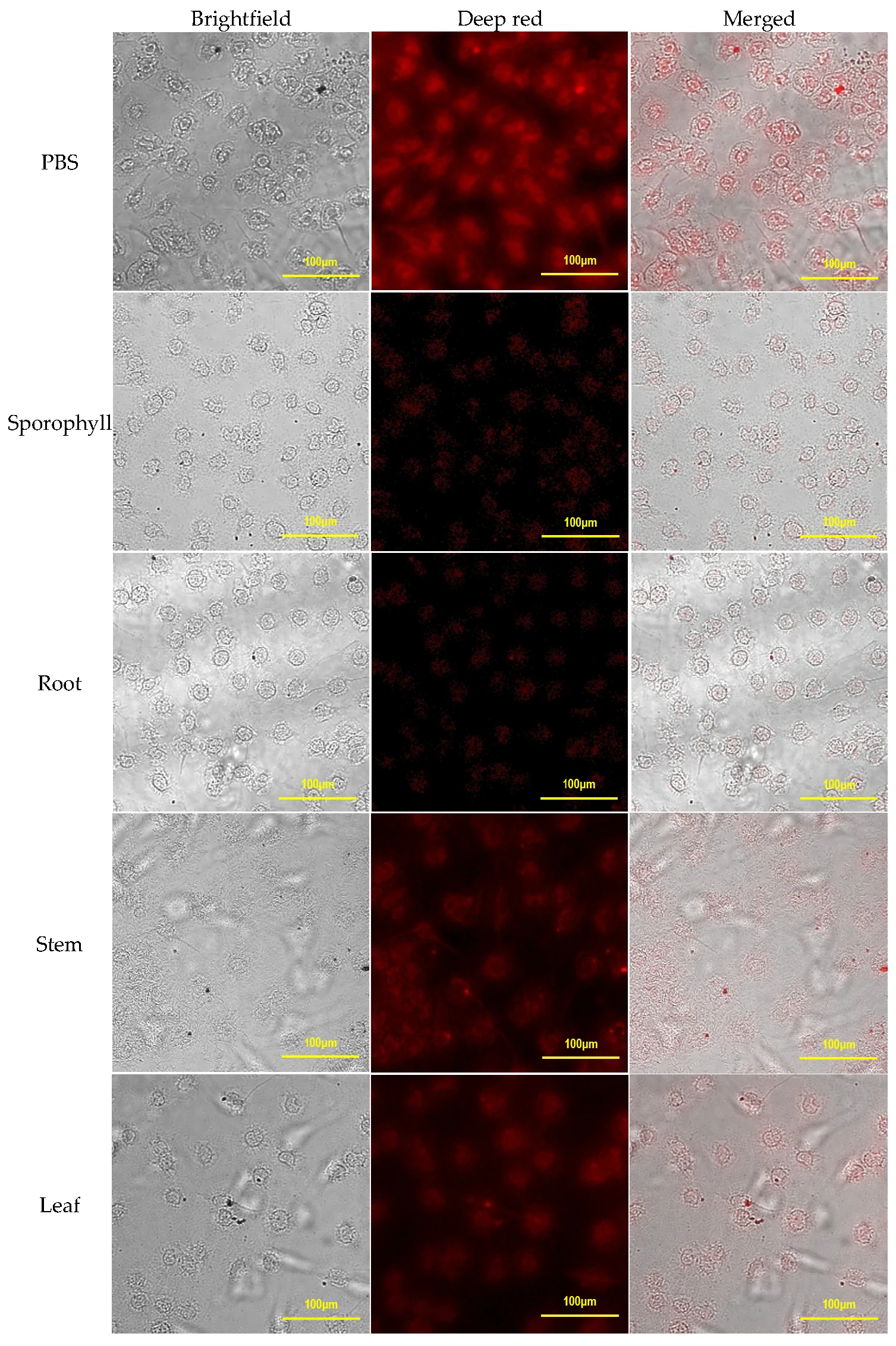
ROS generation in UVB-irradiated L929 cells under the high-glucose culture conditions treated with different *Undaria pinnatifida* extracts. Scale bars = 100 μm.

Seaweed-derived bioactive compounds are well-known for their antioxidant and anti-inflammatory properties, which help mitigate ROS levels [56]. This antioxidant activity may be attributed to the redox potential of various polyphenols and flavonoids as well as pigments and vitamins present in UP extracts, which enables them to effectively neutralize ROS levels in cells. In particular, the root extract demonstrated strong antioxidant properties under the high-glucose culture condition used in this study. Together with its α-glucosidase inhibitory activity, these findings indicate that the root extract possesses multiple in vitro biological activities relevant to further functional evaluation. However, the present results do not establish antidiabetic efficacy or direct protection against UVB-induced DNA damage. Moreover, because the normal and high-glucose conditions were established using different basal media, differences between these culture conditions cannot be attributed solely to glucose concentration. ROS generation of L929 cells treated with UP extracts is shown in Figure 10, Figure 11, Figure 12 and Figure S2.

#### 3.5.4. In Vitro Wound-Closure Activity

**Figure 13:**
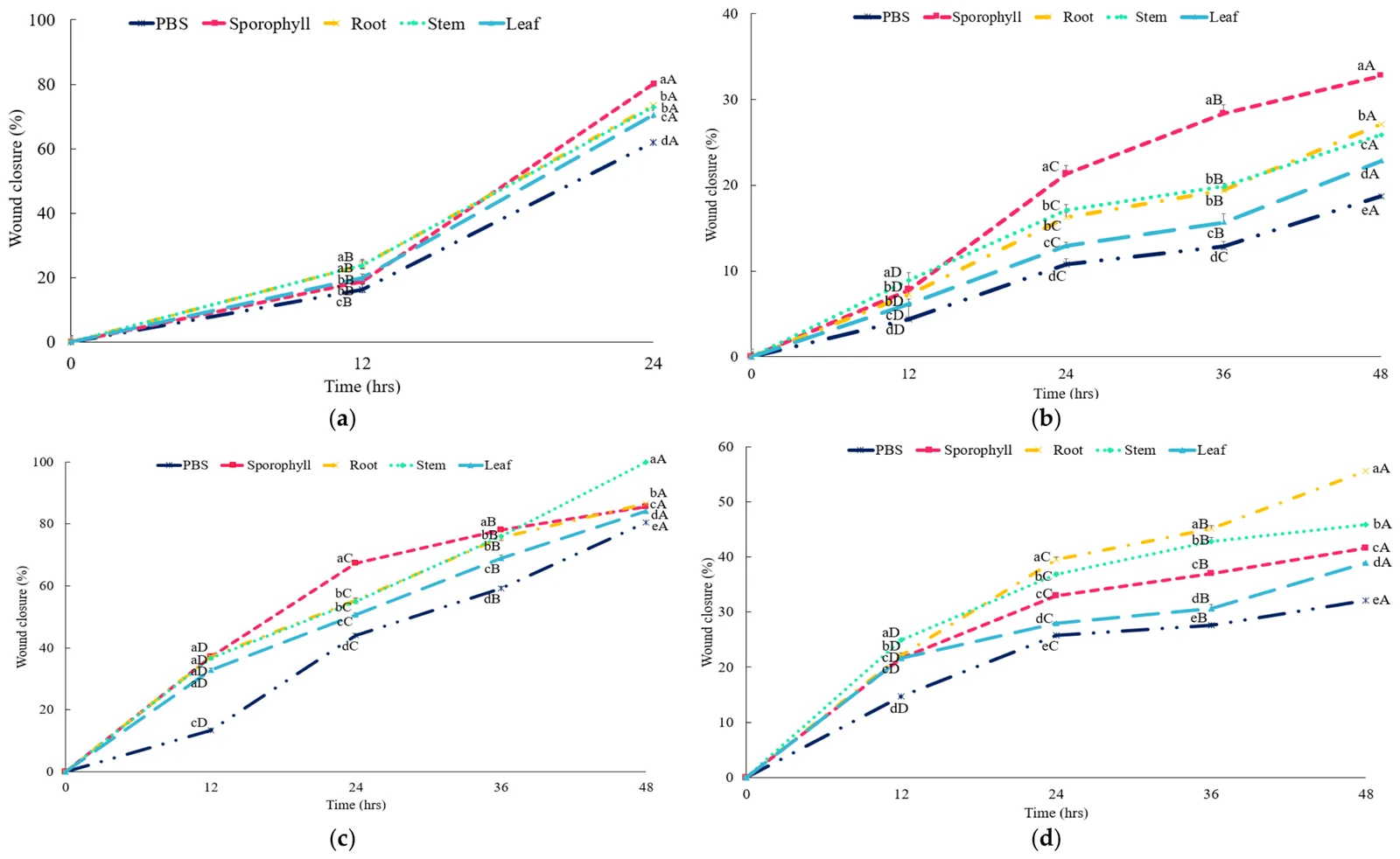
In vitro wound closure (%) of (**a**) non-irradiated L929 cells under the normal culture condition; (**b**) UVB-irradiated L929 cells under the normal culture condition; (**c**) non-irradiated L929 cells under the high-glucose culture condition; and (**d**) UVB-irradiated L929 cells under the high-glucose culture condition treated with different *U. pinnatifida* extracts. Data are presented as the mean ± SD (n = 5). The effects of treatment, time, and the treatment × time interaction were analyzed using two-way repeated-measures ANOVA. Duncan’s multiple range test was used for comparisons among treatments at each time point and among time points within each treatment. Different lowercase letters indicate significant differences among treatments at the same time point, whereas different uppercase letters indicate significant differences among time points within the same treatment (*p* < 0.05).

To evaluate the effects of UP extracts on in vitro wound closure, a scratch assay was performed by mechanically creating wounds in a confluent monolayer of L929 cells. In non-irradiated cells cultured under normal conditions, complete closure was observed within 36 h for all treatments. However, UP-treated cells showed significantly greater wound closure, achieving 70–80% closure at 24 h compared to 60% for untreated cells (Figure 13a). Among the treatments, sporophyll extract showed the greatest wound closure, followed by root, stem, leaf, and untreated groups. Similarly, Premarathna et al. [57] reported improved cell proliferation and migration activities in L929 cells treated with an aqueous extract from the brown alga *Sargassum ilicifolium*. In vivo, albino mice treated with a topical application of the aqueous extract of *S. ilicifolium* exhibited faster wound healing rates compared to the untreated group.

On the other hand, UVB-irradiated cells showed slower wound closure compared to non-irradiated cells, indicating an inhibitory effect of UVB exposure on the in vitro wound-closure response (Figure 13b). After 48 h, untreated cells exhibited an 18% closure rate, while cells treated with UP extracts achieved closure rates of 22–32%, with sporophyll extract showing the greatest wound closure, followed by root, stem, leaf, and untreated group. Jing et al. [58] reported that fucoidan, predominantly found in sporophyll, exhibits antiphotoaging properties against UV irradiation. Their study demonstrated that UP fucoidan mitigates cellular aging and reduces mitochondrial dysfunction in UV-exposed skin.

High-glucose conditions can impair cellular processes involved in wound closure and increase oxidative stress and inflammatory responses [59,60]. This prolonged wound healing may also result in complications such as infections, which, in severe cases, can lead to amputations. In this study, cells cultured under the high-glucose condition showed slower wound closure, requiring 48 h for complete or near-complete closure compared with 36 h under the normal culture condition. Notably, only cells treated with UP stem extract achieved full wound closure within 48 h. However, other UP extracts (sporophyll, root, and leaf) also demonstrated significantly greater scratch closure, ranging from 84.25% to 86.58%, compared to the untreated group, which showed a closure rate of 80.42% (Figure 13c). These findings are consistent with previous reports indicating the potential of brown algal polysaccharides to influence processes associated with tissue repair.

**Figure 14:**
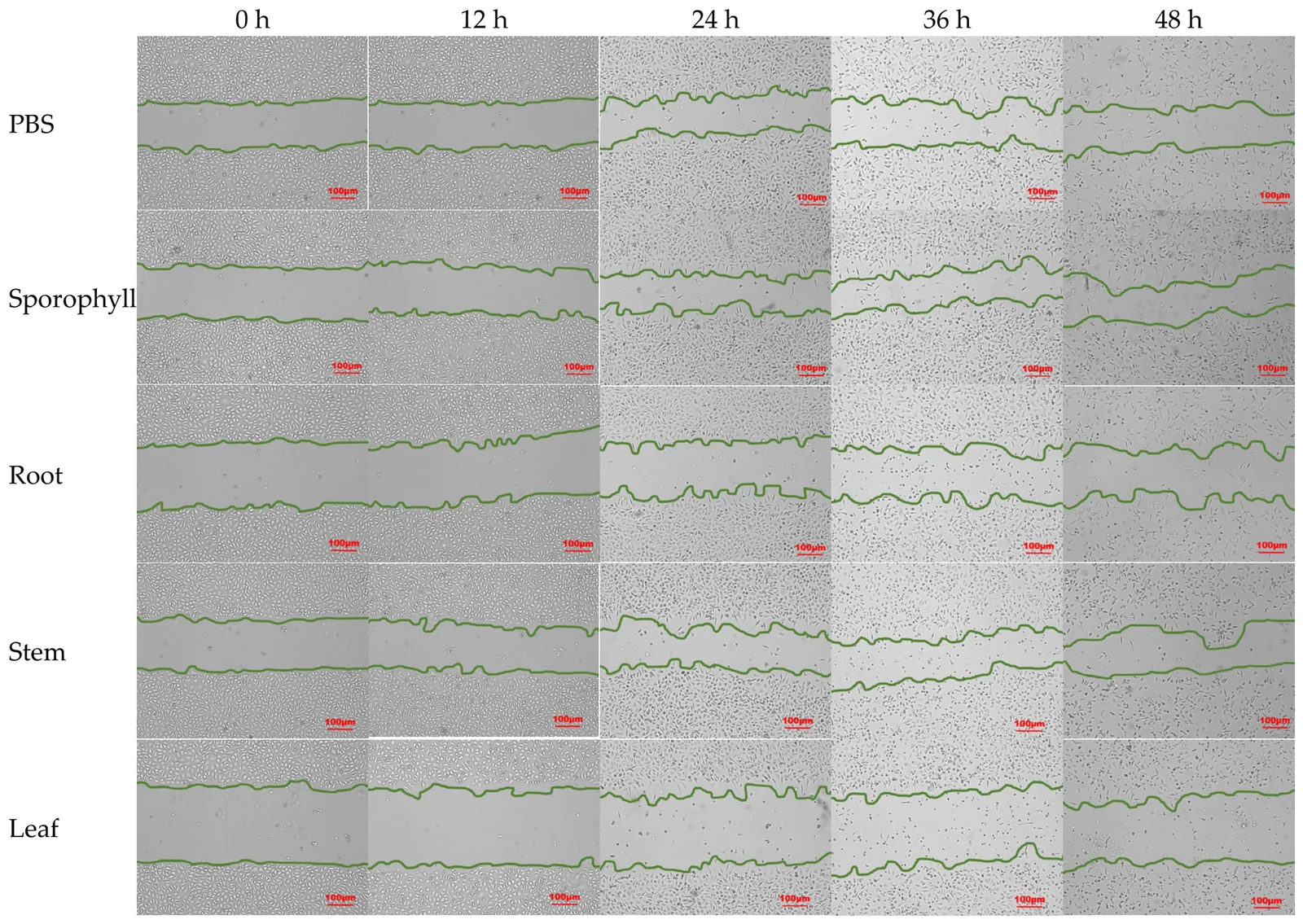
Representative images of in vitro scratch closure in UVB-irradiated L929 cells under the normal culture condition following treatment with *U. pinnatifida* extracts at 0, 12, 24, 36, and 48 h.

**Figure 15:**
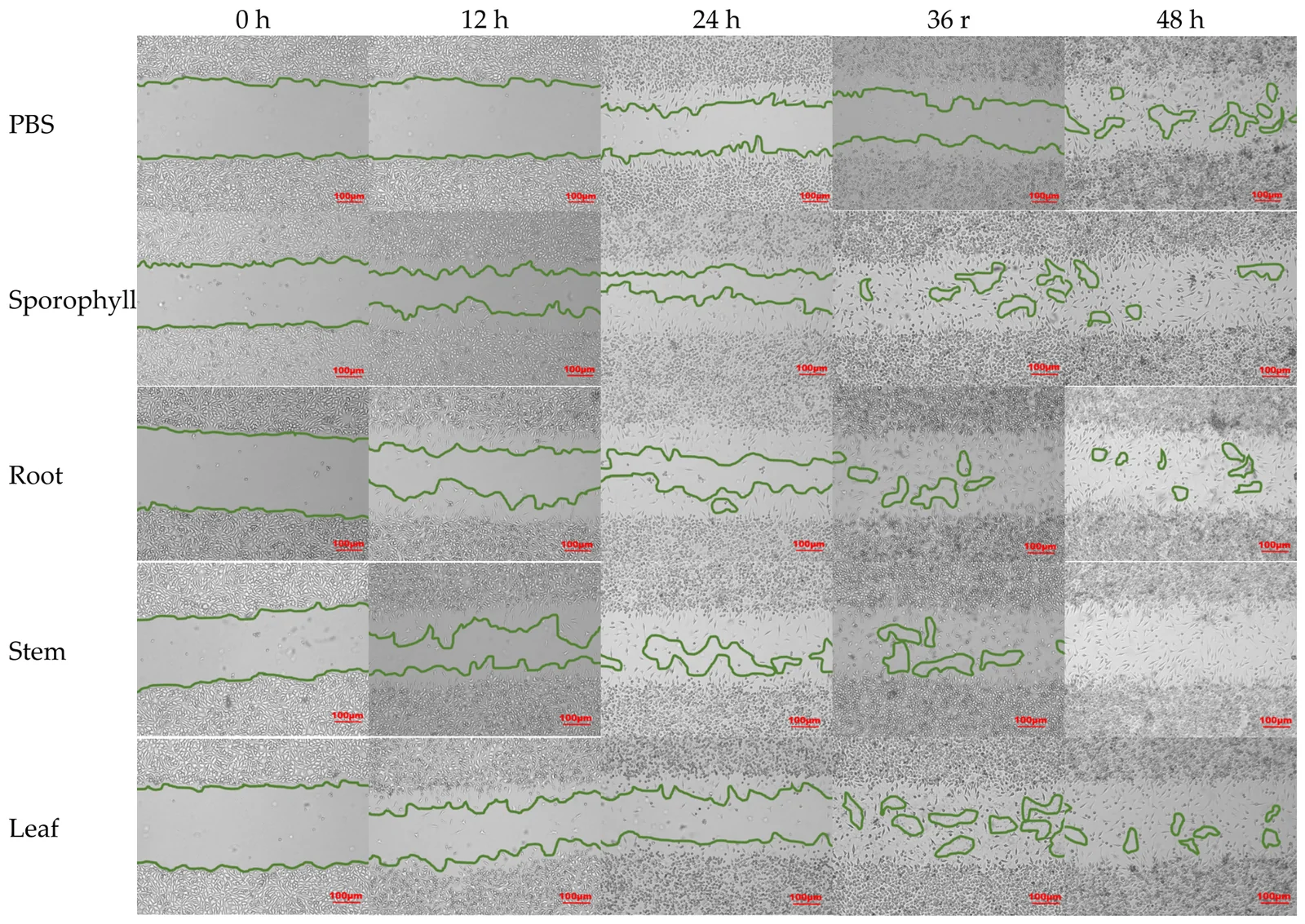
Representative images of in vitro scratch closure in non-irradiated L929 cells under the high-glucose culture condition following treatment with *U. pinnatifida* extracts at 0, 12, 24, 36, and 48 h.

**Figure 16:**
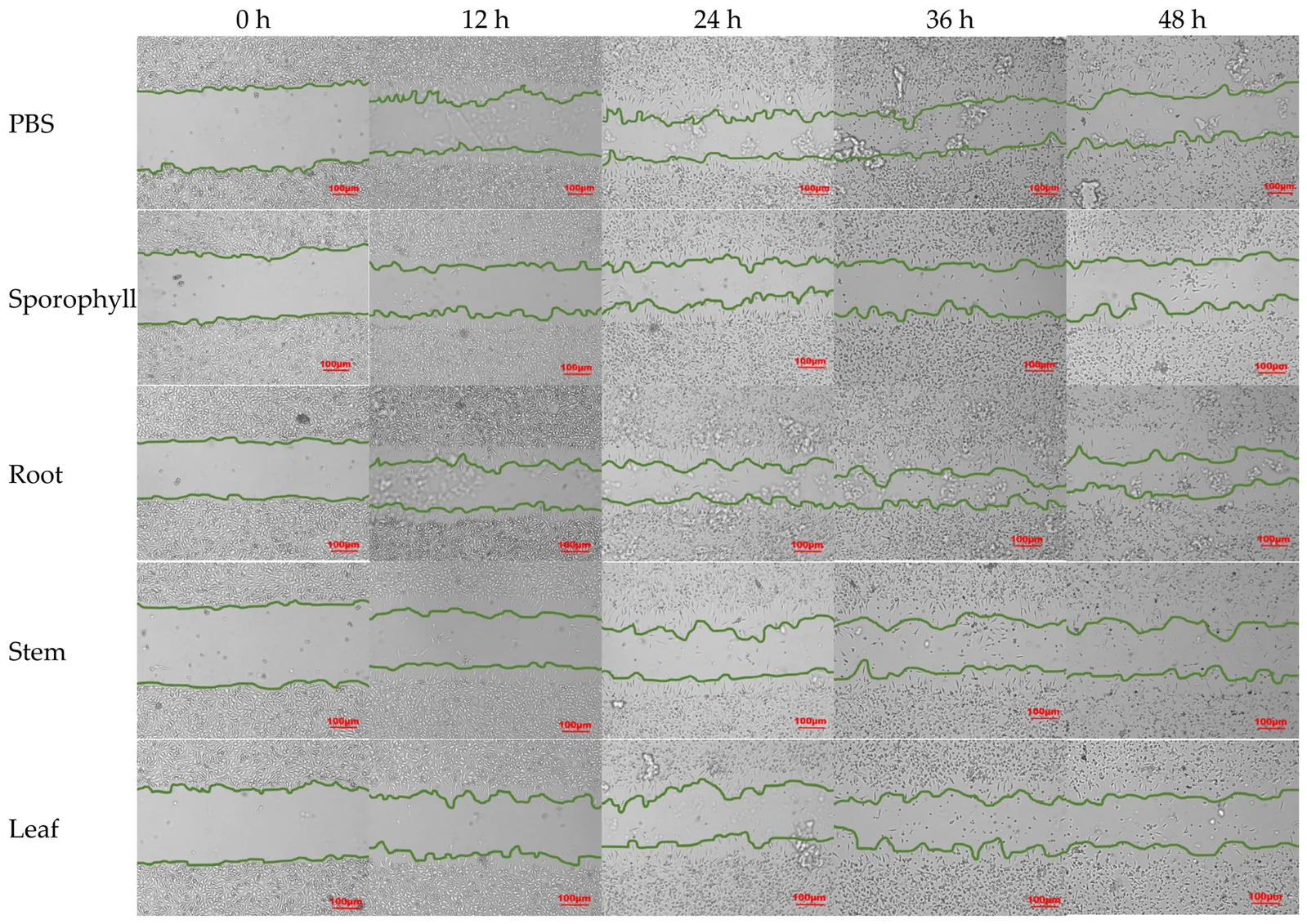
Representative images of in vitro scratch closure in UVB-irradiated L929 cells under the high-glucose culture condition following treatment with *U. pinnatifida* extracts at 0, 12, 24, and 36 h.

Significantly greater in vitro wound closure (*p* < 0.05) was also observed in UVB-irradiated cells cultured under the high-glucose condition and treated with UP extracts than in the untreated group. Root extract was the most effective, with a closure rate of 55.62% after 48 h, followed by stem, sporophyll, and leaf extracts (Figure 13d). UVB-irradiated cells cultured under the high-glucose condition showed lower wound-closure rates, ranging from 32.16% to 55.62% after 48 h, compared to non-irradiated cells cultured under the high-glucose condition. According to Chen et al. [27] high glucose conditions in cells exposed to UVB radiation can lead to increased reactive oxygen species (ROS) levels and cyclobutane pyrimidine dimers (CPD) formation in keratinocytes, which may also heighten their susceptibility to UVB-induced apoptosis. However, CPD formation or other direct markers of DNA damage were not measured in the present study. Therefore, the current findings cannot establish whether UP extracts directly protect cells against UVB-induced DNA damage. Representative images of in vitro wound closure in L929 cells treated with UP extracts are shown in Figure 14, Figure 15, Figure 16 and Figure S3.

It should be noted that scratch closure in a confluent fibroblast monolayer can result from both cell migration and proliferation. Because proliferation was not independently inhibited or quantified in the present study, the observed changes cannot be attributed exclusively to cell migration. Moreover, this in vitro assay does not reproduce the complex inflammatory, vascular, and extracellular matrix processes involved in wound healing in vivo. Accordingly, the present findings are interpreted as effects on in vitro wound closure rather than direct evidence of enhanced cell migration or wound-healing efficacy.

*U. pinnatifida* is rich in bioactive compounds, including fucoxanthin, phlorotannins, flavonoids, and various polysaccharides, all of which are known to exhibit biological activities potentially relevant to wound repair. Ferreira et al. [61] demonstrated that phlorotannin extracted from UP exhibits potent antioxidant, antibacterial, and anti-inflammatory activities, effectively reducing inflammation-induced damage in RAW 264.7 cells. In another study, UP-derived phlorotannin incorporated into electrospun wound dressings showed strong antimicrobial properties without exhibiting cytotoxic effects [62]. These previous findings, together with the present results, suggest that bioactive constituents of UP may contribute to the observed in vitro wound-closure responses. However, the scratch assay used in the present study represents a simplified in vitro model in which closure of the scratched area may result from both cell migration and proliferation. Because these two processes were not independently distinguished in the present experimental design, the increased scratch closure cannot be attributed specifically to enhanced cell migration. Moreover, this assay does not reproduce the complex inflammatory, vascular, and extracellular matrix processes involved in wound healing in vivo. In addition, L929 cells are a murine fibroblast cell line and therefore do not fully represent the biological responses of human skin. Accordingly, the present findings should be interpreted as evidence of enhanced in vitro wound closure under the experimental conditions used rather than as direct evidence of wound-healing efficacy in humans. Further studies using mechanistic assays, human skin-derived cells, and appropriate in vivo models are required to establish the potential relevance of UP extracts to wound-healing applications.

## 4. Conclusions

This study comparatively evaluated 80% ethanol extracts from the leaf and underutilized parts (sporophyll, root, and stem) of *U. pinnatifida* using cell-free enzyme assays and L929 fibroblast-based assays under non-irradiated and UVB-irradiated conditions in normal- and high-glucose culture media. Among the tested extracts, the root extract generally exhibited the strongest inhibitory activities against tyrosinase, hyaluronidase, elastase, collagenase, α-glucosidase, and xanthine oxidase and produced the greatest reduction in intracellular ROS. At 50 μg/mL, the extracts maintained MTS metabolic responses at or above those of the untreated control, while live/dead staining indicated low levels of cell death. The extracts also enhanced in vitro scratch closure relative to untreated cells under several experimental conditions.

These findings demonstrate measurable in vitro biological activities of both the commonly utilized leaf and underutilized parts of *U. pinnatifida*, with the root showing particularly promising activity across several experimental endpoints. However, the cell-free enzyme assays do not directly demonstrate cellular ECM remodeling or anti-aging, anti-wrinkle, or antidiabetic efficacy, and the scratch assay represents in vitro wound closure rather than wound healing in vivo. Direct markers of UVB-induced DNA damage were not evaluated; therefore, the present findings do not establish direct protection against UVB-induced genomic damage. Moreover, because different basal media were used for the normal and high-glucose culture conditions, differences between these conditions cannot be attributed solely to glucose concentration. The use of a single extract concentration (50 μg/mL) in the cell-based assays also limits conclusions regarding concentration-dependent responses. Furthermore, because L929 cells are derived from mice, the present cell-based findings cannot be directly extrapolated to human skin responses. Additional studies using chemically standardized extracts, broader concentration ranges, controlled glucose conditions using the same basal medium, human skin-derived cells, and specific mechanistic and DNA-damage endpoints are required to confirm these activities and establish their relevance to skin-health or cosmetic applications. Nevertheless, the present findings provide a basis for further investigation of underutilized *U. pinnatifida* parts as potential value-added functional materials.

## Figures and Tables

**Table 1:** α-Glucosidase and xanthine oxidase inhibitory activities (IC_50_) of different UP extracts.

Samples	α-Glucosidase Inhibitory Activity IC_50_ (mg/mL)	Xanthine Oxidase Inhibitory Activity IC_50_ (μg/mL)
Acarbose ^1^	1.39 ± 0.07 ^c^	-
Allopurinol ^2^	-	0.40 ± 0.02 ^e^
Sporophyll	0.46 ± 0.00 ^d^	1.14 ± 0.06 ^ab^
Root	0.22 ± 0.01 ^e^	0.65 ± 0.05 ^d^
Stem	3.25 ± 0.18 ^a^	0.87 ± 0.00 ^c^
Leaf	1.88 ± 0.05 ^b^	1.39 ± 0.17 ^a^

^1^ Arcarbose was used as the positive control for measuring α-glucosidase inhibition. ^2^ Allopurinol was used as the positive control for measuring xanthine oxidase inhibition. Different lowercase superscript letters within the same column indicate significant differences among the four *U. pinnatifida* extracts according to one-way ANOVA followed by Duncan’s multiple range test (*p* < 0.05).

## Data Availability

The original contributions presented in this study are included in the article/supplementary material. Further inquiries can be directed to the corresponding author.

## Supplementary Materials

The following supporting information can be downloaded at: https://www.mdpi.com/article/10.3390/foods15193571/s1. Figure S1: Live and dead staining of non-irradiated L929 cells under normal conditions treated with different *U. pinnatifida* extracts. Green fluorescence indicates live cells, while red fluorescence indicates dead cells; Figure S2: ROS generation in non-irradiated L929 cells under normal conditions treated with different *U. pinnatifida* extracts. Scale bars = 100 μm; Figure S3: Representative images of in vitro scratch closure in non-irradiated L929 cells under the normal culture condition following treatment with *U. pinnatifida* extracts at 0, 12, 24, and 36 h.
