# Supplementary material for: Comparative Evaluation of Undaria pinnatifida Anatomical Parts for Skin-Related Functional Properties in UVB-Irradiated L929 Fibroblasts Under Normal and High-Glucose Culture Conditions
Source: Foods. 2026 Oct 8;15(19):3571. doi: 10.3390/foods15193571 (PMC13665017; doi:10.3390/foods15193571)
Supplement: Supplementary file 1 [file foods-15-03571-s001.zip › foods-4490976-supplementary.pdf]

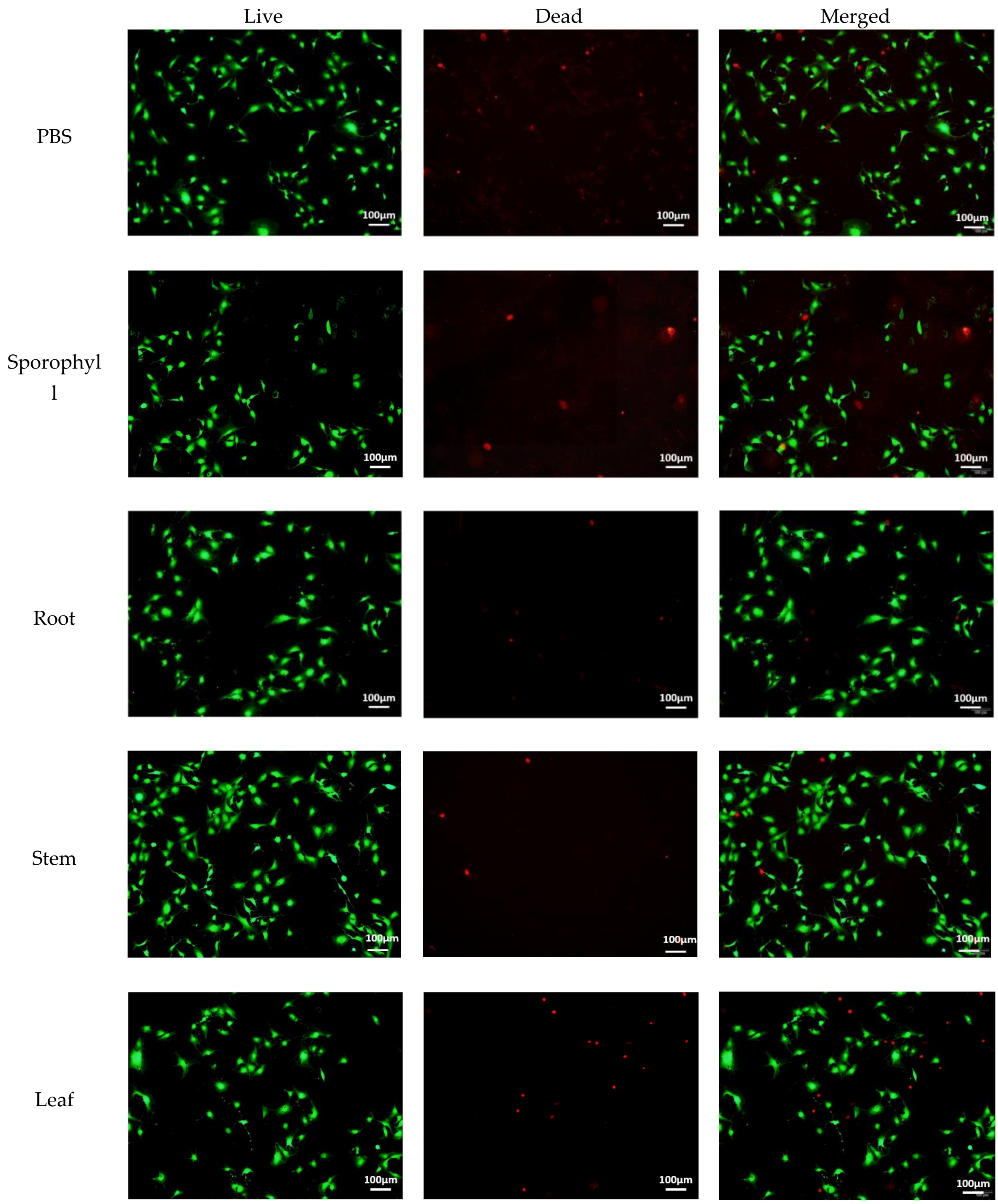

Figure S1. Live and dead staining of non-irradiated L929 cells under normal conditions treated with different *U. pinnatifida* extracts. Green fluorescence indicates live cells, while red fluorescence indicates dead cells.

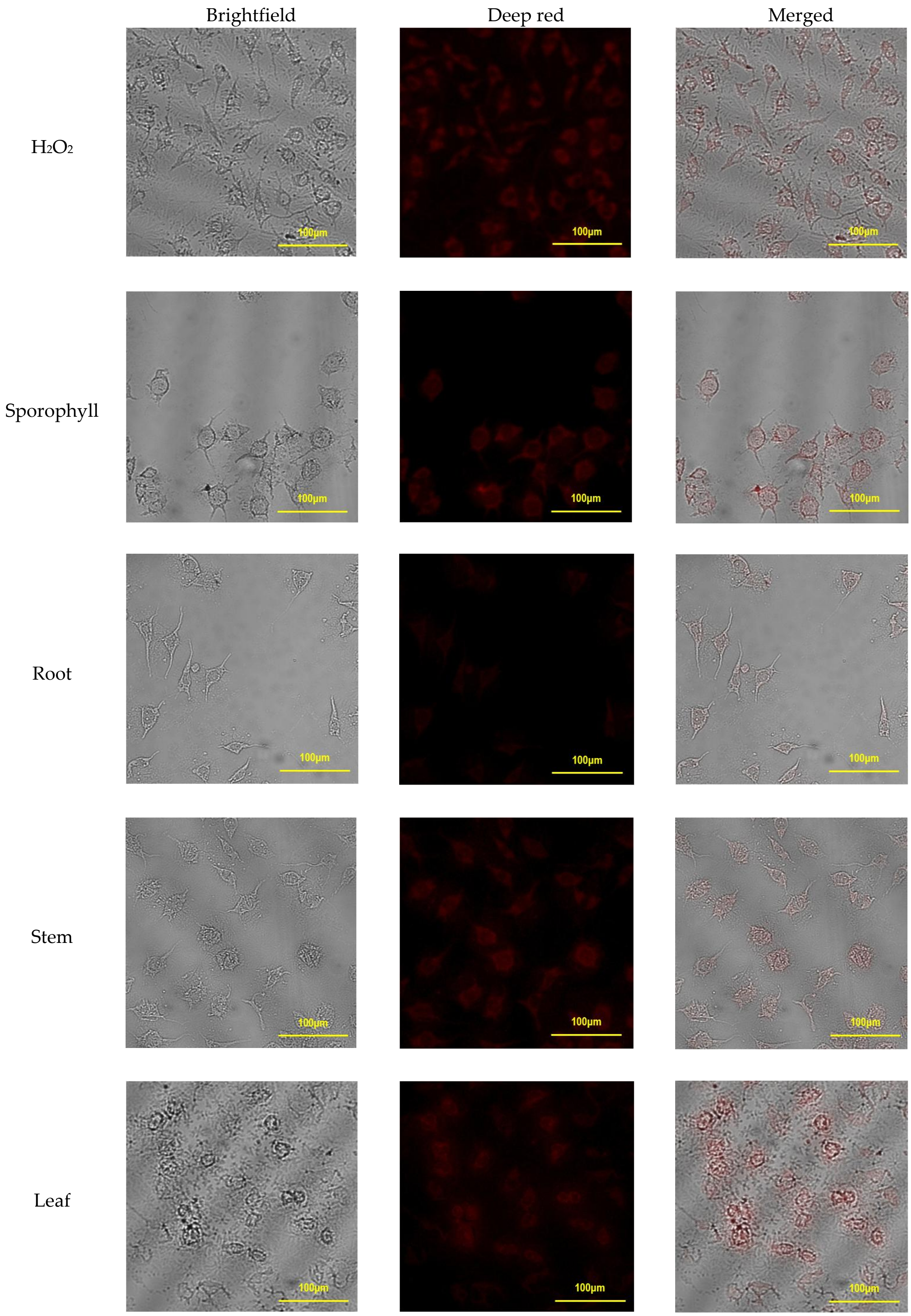

Figure S2. ROS generation in non-irradiated L929 cells under normal conditions treated with different *U. pinnatifida* extracts. Scale bars = 100 μm.

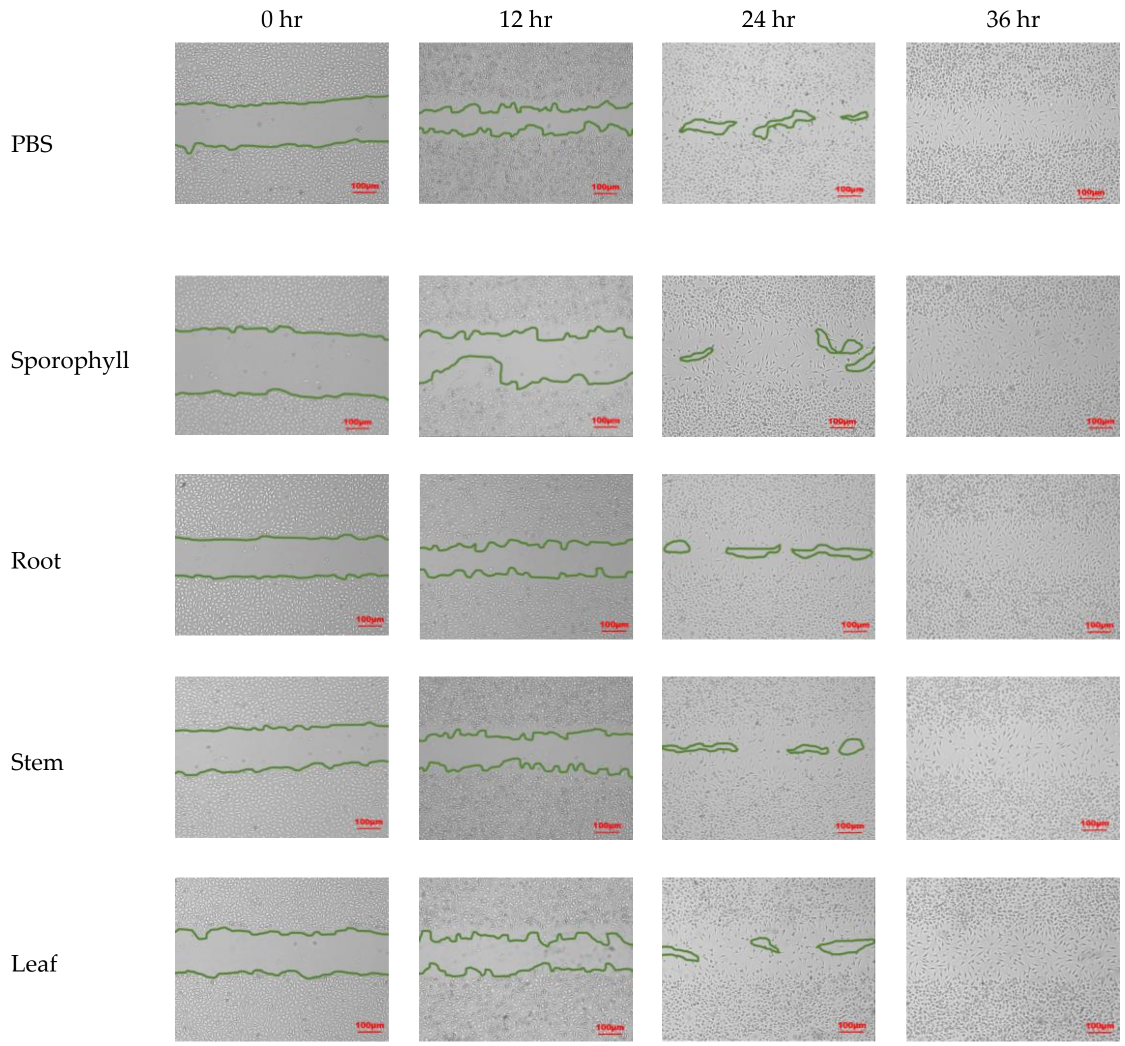

Figure S3. Representative images of in vitro scratch closure in non-irradiated L929 cells under the normal culture condition following treatment with *U. pinnatifida* extracts at 0, 12, 24, and 36 hr.
